# A Single‐Cell Transcriptomic Atlas of the Ovine Rumen Microbiome Characterizes Lineage‐Specific Metabolic Shifts Associated with Host Heat Tolerance

**DOI:** 10.1002/advs.76152

**Published:** 2026-06-15

**Authors:** Sanbao Zhang, Qinyang Jiang, Junjie Ma, Jianwei Zou, Fan Wang, Feifei Lv, Yanna Huang, Yongcheng Wang, Ziye Xu

**Affiliations:** ^1^ Department of Laboratory Medicine of The First Affiliated Hospital & Liangzhu Laboratory Zhejiang University School of Medicine Hangzhou China; ^2^ Guangxi Key Laboratory of Animal Breeding Disease Control and Prevention College of Animal Science and Technology Guangxi University Nanning China; ^3^ Zhejiang Key Laboratory of Clinical In Vitro Diagnostic Techniques Hangzhou China

**Keywords:** heat tolerance, intra‐species heterogeneity, metabolic shift, rumen microbiome, single‐cell transcriptomics

## Abstract

The adaptation of complex, host‐associated microbiomes to environmental perturbations is a critical determinant of ecosystem stability and resilience to climate change, as exemplified in ruminants. While single‐microbe RNA sequencing advances community interrogation, complex microbial cell walls severely constrain unbiased single‐cell transcriptomic profiling in the rumen. In this study, we developed an optimized 25 min time‐resolved enzymatic lysis strategy using smRandom‐seq to map the sheep rumen microbiome at single‐cell resolution. By profiling 60 748 cells across 21 samples, we captured previously intractable lineages, resolving the transcriptional states of 213 genera and 662 species, achieving a physiologically relevant 0.303% recovery of methanogenic archaea. Unsupervised clustering partitioned the ecosystem into seven cross‐species functional clusters, uncovering a spatial coupling between microbial lifestyle and metabolic specialization. Applying this framework to a model of host thermal adaptation demonstrated that host resilience was associated with rapid transcriptional activation of key energy‐metabolism clusters. Notably, a lineage‐specific metabolic shift toward a glycolytic phenotype in *Anaerovibrio lipolyticus* contributes to a compensatory “nutritional sparing” effect associated with host resilience. This dataset provides a foundational resource for rumen microbial ecology and establishes a technical framework for dissecting phenotypic plasticity within complex microbiomes.

## Graphical abstract

An optimized single‐cell transcriptomic framework profiles over 60 000 cells to map the ovine rumen microbiome, partitioning the ecosystem into seven cross‐species functional clusters. In heat‐resistant hosts, a lineage‐specific metabolic shift in *Anaerovibrio lipolyticus* toward a highly glycolytic phenotype contributes to a “nutritional sparing” effect, bolstering host resilience to environmental thermal stress.

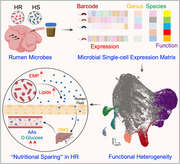

## 1 Introduction

Microbial communities play a crucial role in the adaptation of both livestock and human health to global climate change and the increasing frequency of extreme heat events [1, 2, 3], with ruminants serving as a prime example, where microbial activity generates significant metabolic heat during fermentation [4]. The rumen microbiome, a complex ecosystem of trillions of symbionts, acts as an essential “second genome” that regulates host nutrient acquisition, immune modulation, and physiological homeostasis [5, 6]. While recent studies have linked rumen microbial community shifts to host stress responses, the specific mechanistic pathways by which the microbiome confers thermotolerance remain largely obscure.

Current understanding of the rumen ecosystem relies heavily on “bulk” metagenomics and metatranscriptomics. While powerful for outlining taxonomic composition and potential gene inventories, these community‐averaged approaches treat all cells within a species as functionally identical, inherently masking intrinsic phenotypic heterogeneity [7, 8]. In fluctuating environments like the heat‐stressed rumen, bacteria often employ “bet‐hedging” strategies by differentiating into distinct functional subpopulations (e.g., rapid growth vs. stress persistence) to ensure community survival [8, 9]. Traditional bulk sequencing inevitably averages out these critical dynamics, leaving the true drivers of ecosystem resilience hidden within data “noise,” necessitating single‐cell resolution [10, 11, 12]. To address this, our team developed and applied high‐throughput single‐cell transcriptomics to the bovine rumen, characterizing 2534 microbial species [8]. However, this initial study encountered methodological biases that resulted in the complete absence of archaeal profiles. This absence stems from the unique pseudomurein cell walls of rumen methanogenic archaea, which are notoriously resistant to conventional enzymatic lysis [13, 14]. Standard in situ RNA capture inherently struggles with a strict structural trade‐off that introduces a systematic capture bias, wherein lysis protocols gentle enough to protect fragile Gram‐negative bacteria and labile transcripts inevitably fail to penetrate robust archaeal envelopes [15, 16]. Because of this technical blind spot, a comprehensive single‐microbe atlas of the rumen ecosystem has yet to be established, leaving the micro‐scale mechanisms driving host thermotolerance entirely unexplored.

To address these challenges, we optimized the smRandom‐seq pipeline with a time‐resolved enzymatic lysis strategy tailored for the ovine rumen matrix [8, 17, 18]. Using this approach, we profiled 60 748 individual cells from a sheep model of thermal adaptation to construct a high‐resolution transcriptomic atlas. Rather than simply cataloging taxonomic diversity, this dataset partitions the rumen ecosystem into seven specialized functional clusters. Importantly, we found that despite a largely conserved community structure, specific energy‐harvesting clusters exhibit distinct transcriptional changes associated with host thermotolerance. One example of this plasticity is the lineage‐specific metabolic shift in *Anaerovibrio lipolyticus*. This population shows a shift from a basal translational state toward a more glycolysis‐associated transcriptional profile, which may contribute to host metabolic adaptation under heat stress. Together, these findings provide a foundational resource for rumen microbial ecology and a framework for dissecting in situ phenotypic plasticity in complex microbiomes.

## 2 Results

### 2.1 A High‐Resolution Single‐Cell Atlas of the Sheep Rumen Microbiome

**FIGURE 1:**
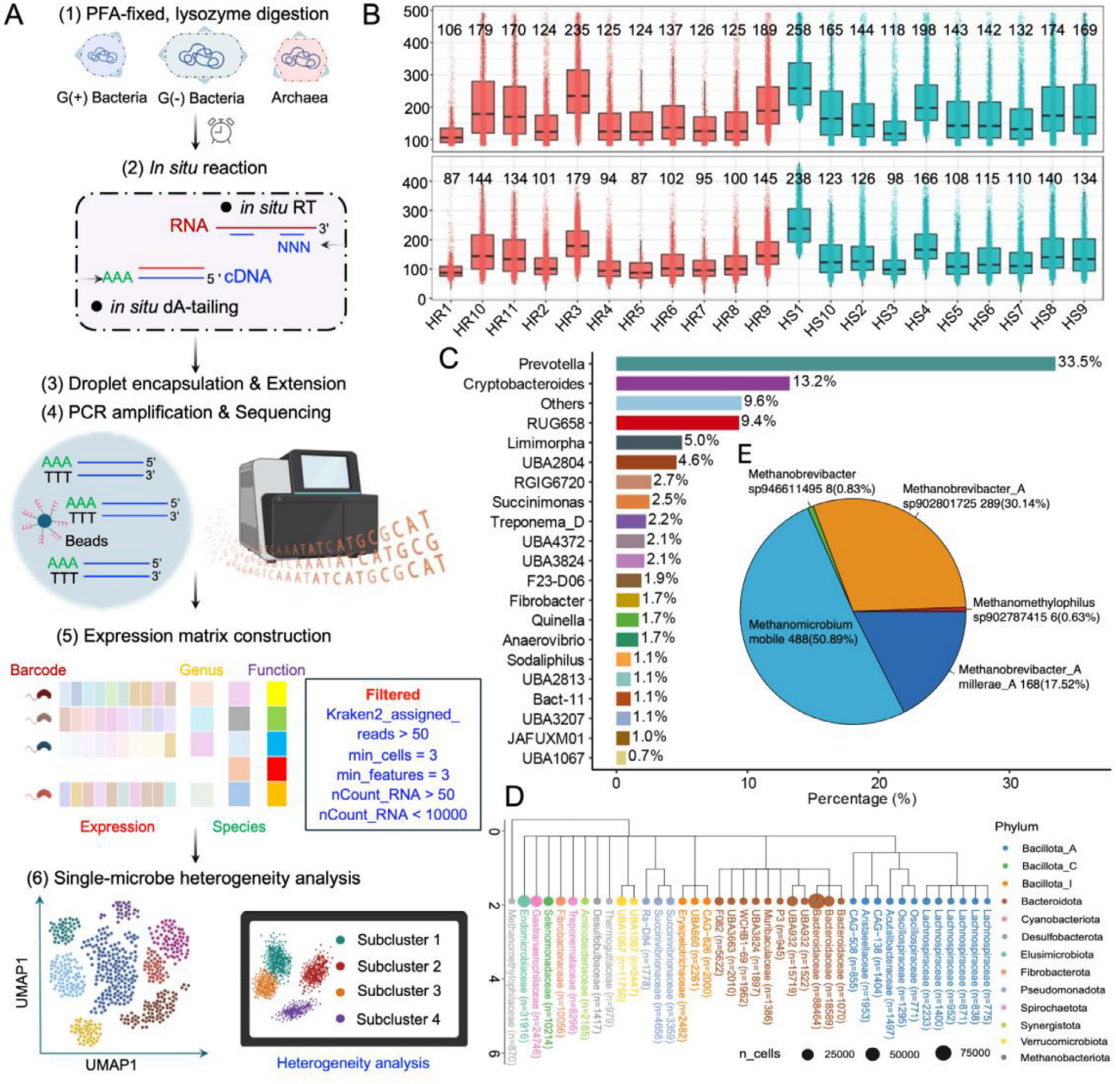
Construction of a high‐resolution single‐cell atlas of the Hu sheep rumen microbiome. (A) Schematic overview of the smRandom‐seq pipeline. (B) Quality control metrics of the single‐cell library. Violin plots display the distribution of Unique Molecular Identifiers (UMIs, top) and detected genes (bottom) per cell across Heat‐Resistant (HR, *n* = 11) and Heat‐Sensitive (HS, *n* = 10) groups. (C) Genus‐level taxonomic distribution of the bacterial community. (D) Phylogenetic reconstruction of the rumen microbiome. The tree highlights the taxonomic diversity of 60 748 annotated cells colored by phylum. Dot size is proportional to the number of reads assigned to each node. (E) Taxonomic composition and read distribution of classically cultured methanogenic archaea.

To overcome the inherent resolution limits of bulk metatranscriptomics in capturing micro‐ecosystem heterogeneity, we optimized our single‐microbe RNA sequencing (smRandom‐seq) pipeline specifically for the highly complex ovine rumen matrix. This platform uniquely integrates in situ enzymatic lysis, reverse transcription, and polyadenylation, followed by single‐cell barcoding within microfluidic droplets, thereby maximizing RNA capture efficiency while preserving single‐cell identity (Figure 1A). To address the pervasive challenge of cell wall heterogeneity and overcome the recalcitrant envelopes of methanogenic archaea, we engineered a time‐resolved, matrix‐specific permeabilization window coupling Tween‐20 treatment with tailored enzymatic digestion (see Methods). Specifically, time‐course evaluations from 15 to 35 min indicated that 25 min was the optimal digestion duration. DAPI staining of pure *Methanobacterium ruminantium* and complex rumen samples verified that the 25 min treatment enables nucleic acid accessibility while preserving cell morphology (Figure S1A). Quantitative PCR further supported this optimum, showing insufficient archaeal DNA recovery at 15 min (Ct ∼ 20) and maximum release at 25 min (Ct ∼ 15). Extending lysis to 35 min induced nucleic acid leakage from fragile rumen taxa and increased the Ct from 14 to 16 (Figure S1B). The defined 25 min protocol therefore effectively breaches archaeal pseudo‐murein walls while preventing community over‐digestion to ensure unbiased multi‐kingdom capture.

We subsequently applied this high‐resolution platform to dissect the microbial mechanisms underlying the adaptation of thermotolerance in sheep. Prior to sequencing, we validated the phenotypic distinctness of our study cohort raised under natural summer heat stress; physiological assessments, including average daily feed intake, revealed a significant divergence between Heat‐resistant (HR) and Heat‐sensitive (HS) individuals (Figures S2 and S3). These significant physiological disparities confirmed the reliability of our genetic selection, providing a robust biological context for characterizing the microbiome at single‐cell resolution. Applying our smRandom‐seq pipeline to 21 rumen microbial samples (10 HS and 11 HR), we generated a comprehensive single‐cell dataset comprising 60 748 high‐quality microbial cells (27 177 from HR and 33 571 from HS hosts). Quality control analysis demonstrated the consistency of our library construction, achieving a mean capture of 156 unique molecular identifiers (UMIs) and 125 genes per cell (Figure 1B). Taxonomic annotation revealed a rich microbial diversity encompassing 213 genera and 662 species (Table S4). At the genus level, the overall community was dominated by *Prevotella* (33.5%) and *Cryptobacteroides* (13.2%) (Figure 1C). Further resolution at the species level revealed that *UBA4372 sp017447625* (20.4%) and *RUG658 sp002448285* (13.3%) were the most predominant taxa, followed by *RGIG6720 sp017525225* (4.9%) and *Prevotella sp900314655* (4.1%) (Figure S4). Beyond accurately recapitulating the canonical composition of the native small ruminant rumen [19, 20, 21], the optimized pipeline demonstrates stable technical fidelity across diverse host ecosystems. Despite inherent host‐driven biological divergence [22, 23] (∼45.05% shared Metagenome‐Assembled Genomes between cattle and sheep [24]), our comparative analysis revealed a highly conserved core microbial atlas with the bovine dataset. Specifically, we consistently identified the 22 shared core bacterial genera, preserving the dominance of fundamental degraders like *Prevotella* (Figure S5A,B). This conserved core microbiota was further quantitatively supported by a moderate positive correlation in their log10‐transformed abundances (Pearson R = 0.581) (Figure S5C).

To validate our optimized pipeline against the naturally low baseline abundance of rumen archaea, we assessed our initial capture efficiency. The pre‐filtered dataset successfully recovered 875 archaeal cells, accounting for 0.303% of the total microbial population (Figure 1D, Table S4). This proportion closely aligns with established in vivo estimates for the rumen ecosystem [24, 25], confirming unbiased sampling. Taxonomic annotation identified seven archaeal species spanning discrete functional clades (Table S4). The community encompassed classically cultured hydrogenotrophic (e.g., *Methanobrevibacter* spp.) and methylotrophic (e.g., *Methanomethylophilus sp902787415*) methanogens, as well as two uncultivated methylotrophic “dark matter” lineages (*SIG5* and *UBA71*) (Figure 1E, Table S4). The successful recovery of these historically recalcitrant lineages confirms that our optimized lysis protocol effectively mitigates systematic capture biases. This represents a substantial technical advancement over our previous bovine study, where archaeal lineages were underrepresented [8]. Ultimately, this approach facilitates culture‐independent, in situ functional characterization, thereby bypassing traditional isolation biases to decode the metabolic heterogeneity of the native microbiome at true single‐cell resolution.

### 2.2 Functional Compartmentalization and Metabolic Coordination of the Rumen Microbiome at Single‐Cell Resolution

**FIGURE 2:**
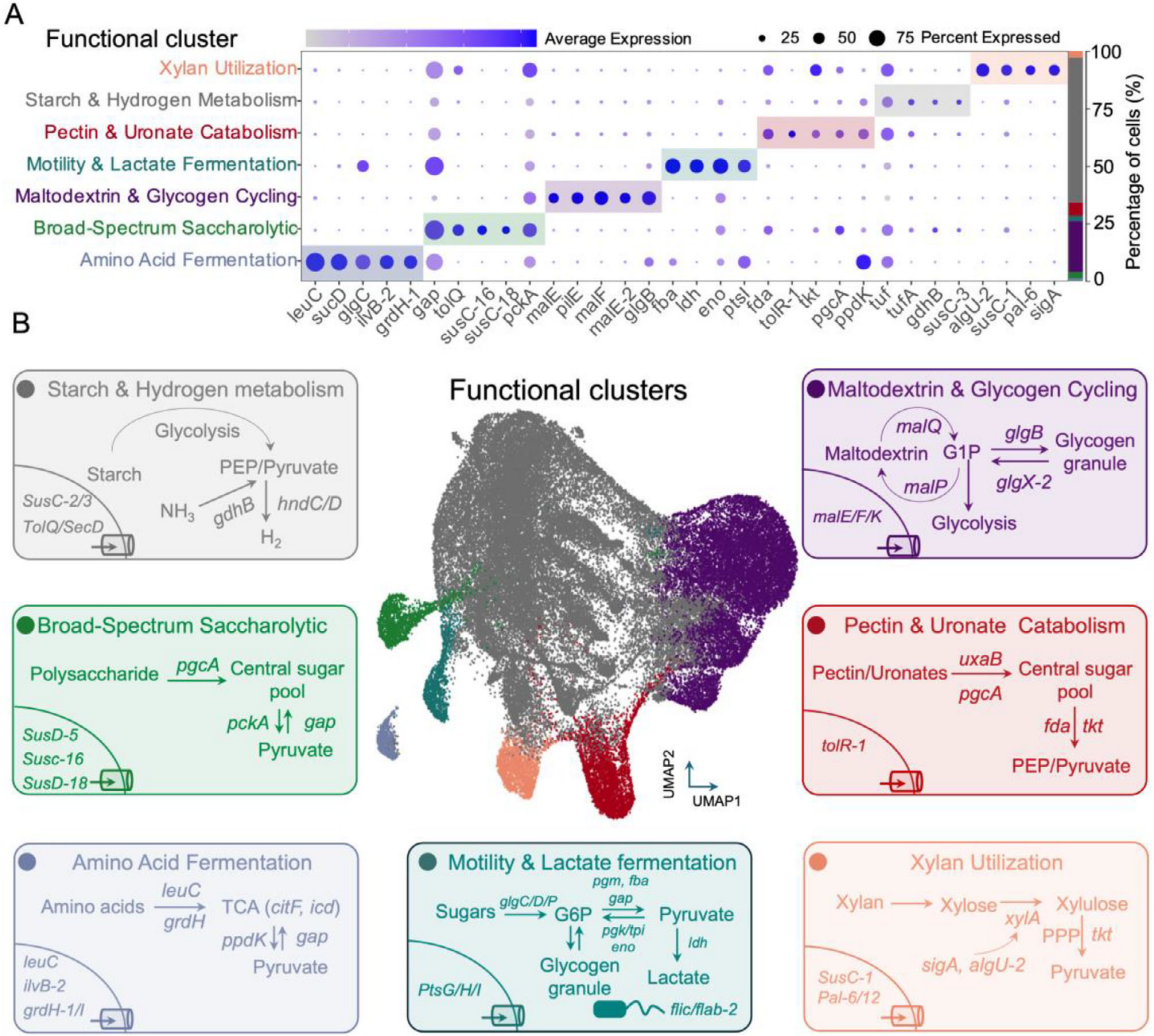
Functional compartmentalization and metabolic orchestration of the rumen microbiome at single‐cell resolution. (A) Characterization of functional clusters and their metabolic signatures. The dot plot visualizes the expression of top discriminatory marker genes (*x*‐axis) across the seven identified functional clusters (left *y*‐axis). The stacked bar chart on the right *y*‐axis, labeled “Percentage of cells (%),” illustrates the relative abundance (proportion) of each functional cluster within the total profiled cell population. Dot size indicates the percentage of cells expressing the gene; color intensity represents the average expression level (scaled log‐normalized counts). (B) Uniform Manifold Approximation and Projection (UMAP) embedding of 60 748 high‐quality microbial cells. The plot reveals the topological landscape of the rumen microbiome, partitioned into seven distinct functional clusters.

To dissect the functional heterogeneity of the rumen ecosystem beyond taxonomic classification, we performed unsupervised clustering on the transcriptomes of 60 748 high‐quality microbial cells. Through a rigorous over‐clustering and merging strategy validated by topological stability assessment (Figure S6), this analysis partitioned the complex community into seven distinct functional clusters, each defined by a unique gene expression signature that is not strictly constrained by taxonomic lineage (Figure 2A, Table S5). The metabolic landscape was overwhelmingly dominated by the Starch & Hydrogen Metabolism (62.95%; 38 240 cells) and Maltodextrin & Glycogen Cycling (22.12%; 13 437 cells) clusters, which together constitute the ecosystem's metabolic backbone. These core clusters were complemented by specialized functional niches, including Pectin & Uronate Catabolism (5.73%; 3479 cells), Xylan Utilization (2.94%; 1785 cells), and Amino Acid Fermentation (1.18%; 718 cells), revealing a highly structured division of labor essential for efficient biomass conversion (Figure 2A).

To investigate the mechanistic basis of these functional states, we identified top discriminatory marker genes (Figure 2B), uncovering a coordinated “uptake‐catabolism‐excretion” cascade that underpins host‐microbe symbiosis. The metabolic trajectory begins with primary nutrient acquisition, where the Broad‐Spectrum Saccharolytic and Xylan Utilization clusters act as “frontline” degraders. These populations are heavily enriched for surface‐localized transport systems, such as Sus‐like nutrient transporters (*susC‐1*, *susC‐16*) and TonB‐dependent receptors (*tolQ*), to facilitate the active sequestration and periplasmic internalization of complex plant polysaccharides. Upon internalization, substrates are channeled into specialized catabolic and storage pathways. Specifically, the Maltodextrin & Glycogen Cycling cluster, characterized by high expression of the maltose transport complex (*malE, malF*) and glycogen branching enzymes (*glgB*), functions to scavenge starch breakdown products and buffer energy via intracellular glycogen reserves, while the Pectin & Uronate Catabolism cluster simultaneously upregulates transketolase (*tkt*) and fructose‐bisphosphate aldolase (*fda*) to shunt uronic acid derivatives into the pentose phosphate pathway. Downstream of these catabolic events, the Motility & Lactate Fermentation cluster drives energy conservation by exhibiting peak expression of glycolytic enzymes, such as glyceraldehyde‐3‐phosphate dehydrogenase (*gap*), enolase (*eno*), and lactate dehydrogenase (*ldh*), thereby rapidly coupling carbon flux to ATP generation and volatile fatty acid (VFA) synthesis. Distinctly, the Amino Acid Fermentation cluster, marked by isopropylmalate isomerase (*leuC*) and succinyl‐CoA synthetase (*sucD*), specializes in the recycling of nitrogenous compounds and the synthesis of branched‐chain amino acids, thus completing the ecosystem's metabolic cycle. This spatially and transcriptionally segregated architecture confirms that the rumen microbiome operates not merely as a mixture of species, but as a multi‐layered digestive organ [26]. In this system, specific cell populations are programmed to transport extracellular nutrients, digest them through niche‐specific enzymatic cascades, and excrete secondary metabolites that fuel ruminant physiology [27].

### 2.3 Single‐Cell Resolution Unmasks Functional Architectures and Phenotypic Plasticity Inaccessible to Bulk Metatranscriptomics

**FIGURE 3:**
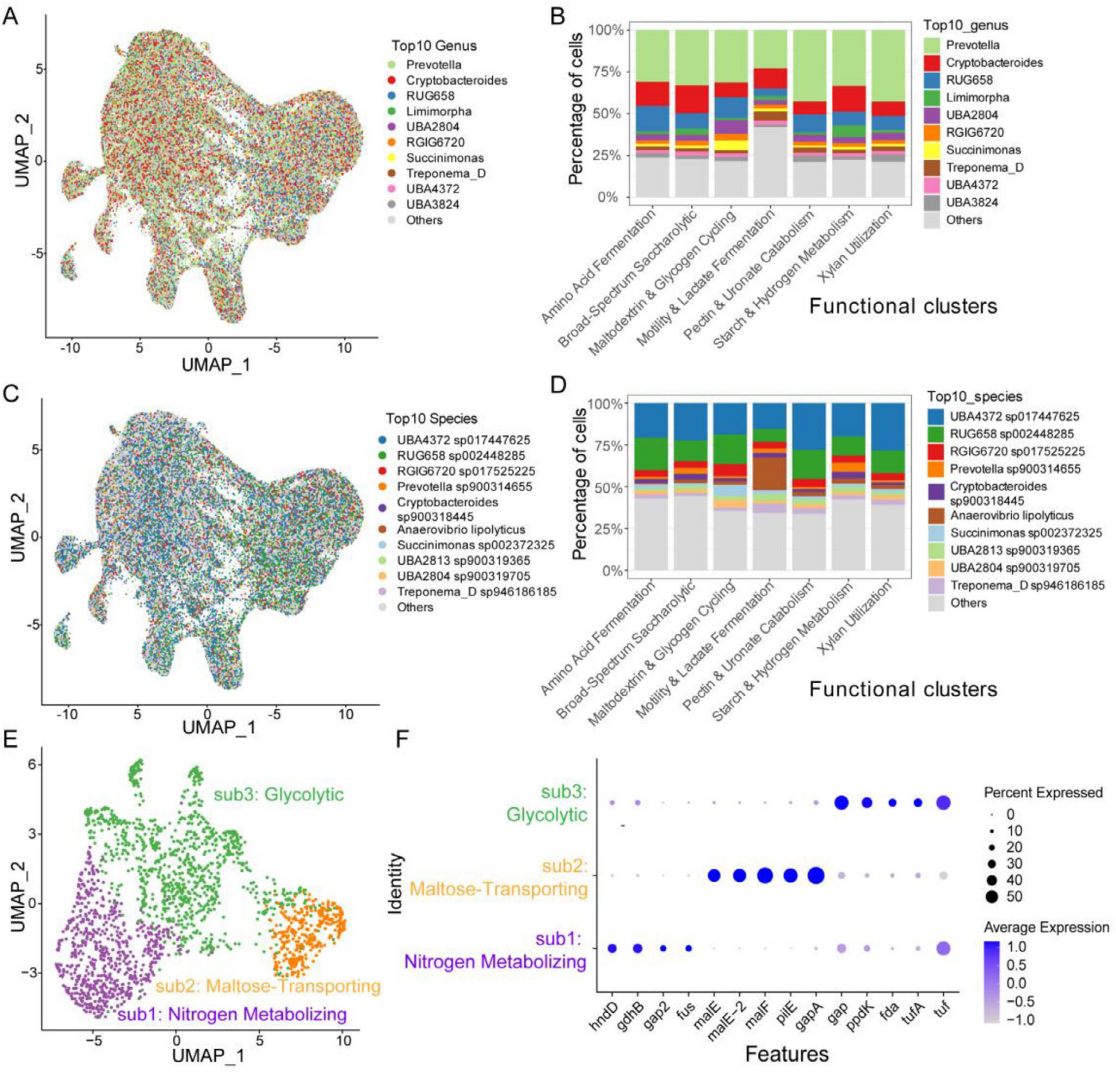
Single‐cell resolution unmasks the spatial‐functional architecture and homeostatic mechanisms of rumen metabolic clusters. (A) UMAP (Uniform Manifold Approximation and Projection) visualization displaying the distribution of the top 10 microbial genera across the identified functional clusters. (B) Bar plots quantifying the relative abundance of these dominant genera. (C) UMAP projection illustrating the distribution of the top 10 microbial species within the ecosystem's functional clusters. (D) Bar plots quantifying the relative abundance of these dominant species. (E) Sub‐clustering of the core xylanolytic taxon *Cryptobacteroides sp900318445* reveals three discrete transcriptional states (Sub‐clusters 1–3). (F) Functional annotation of *Cryptobacteroides sp900318445* sub‐clusters.

Unlike bulk metatranscriptomics, which averages transcriptional outputs across the community, the single‐cell platform allowed the analysis of taxon‐specific functions. For example, proteolytic activity was mapped primarily to *Prevotella*, which constituted 30.9% of the Amino Acid Fermentation cluster (Figure 3A,B, Table S6). We also identified a distinct metabolic pairing where motility and lactate fermentation were driven by *Anaerovibrio lipolyticus*, linking flagellar assembly to the chemotactic pursuit of lipids (Figure 3C,D, Table S7). Moreover, the ecosystem's core Starch & Hydrogen Metabolism was sustained not by standard reference isolates, but by uncultured lineages such as *UBA4372* and *RUG658* (aggregating ∼36% of the cluster), highlighting the limitations of reference‐based metagenomic inferences.

Crucially, our single‐cell framework resolves the dynamic physical and trophic interactions among rumen functional guilds, revealing a structured division of labor rather than a randomized mixture. First, a hierarchical cross‐feeding network drives biomass conversion. Amino acid fermentation provides the essential cellular capital to sustain primary fiber digestion, highlighting an interdependency between nitrogen recycling and carbon breakdown (Figure S7). Second, metabolic compartmentalization is coupled to microbial lifestyle. Primary degrading clusters (e.g., Xylan Utilization; *Cryptobacteroides*) upregulate adhesins and pili to establish an “Adherent” niche on plant matrices (Figure S8). Conversely, secondary fermenters (e.g., Motility & Lactate Fermentation; *Methanobrevibacter*) highly express flagellar pathways, adopting a motile “Planktonic” lifestyle to actively scavenge released intermediates. Finally, this spatial segregation maximizes thermodynamic efficiency and pH homeostasis (Figure S9). Direct cluster‐to‐cluster interactions ensure that sugars and H_2_ released by adherent degraders are immediately consumed by neighboring planktonic satellites. This rapid scavenging minimizes product inhibition and creates a buffered microenvironment, evidenced by the highly stable intracellular pH of adherent cells, protecting acid‐sensitive degraders and collectively maintaining macroscopic rumen homeostasis.

To resolve functional heterogeneity at the intra‐species level, we analyzed the transcriptional subpopulations of the core rumen taxon *Cryptobacteroides sp900318445* (Figure 3E,F, Table S8), identifying three distinct functional states. Sub‐cluster 1 represents a “Nitrogen metabolizing” state, driven by key assimilation and redox genes (*gdhB*, *hndD*), it focuses on active amino acid turnover. Sub‐cluster 2 acts as a “Maltose transporting” energy sink. By upregulating maltose transporters (*malE, malF*, and *malK*) and glycogen cycling enzymes (*glgB, glgX*), this population scavenges transient sugar spikes to build intracellular reserves, which buffer metabolic energy and help stabilize rumen pH. Distinctly, Sub‐cluster 3 exhibits a “Glycolytic” profile, fueling central carbon catabolism through robust expression of core pathway enzymes (*gap, fda* and *tkt*). Together, this segregation reveals an intra‐species “division of labor.” By decoupling episodic carbohydrate storage from sustained glycolysis and nitrogen assimilation, this species optimizes its overall efficiency within the highly fluctuating rumen environment.

### 2.4 Targeted Microbial Transcriptional Rewiring Is Associated with Host Systemic Metabolic Resilience

**FIGURE 4:**
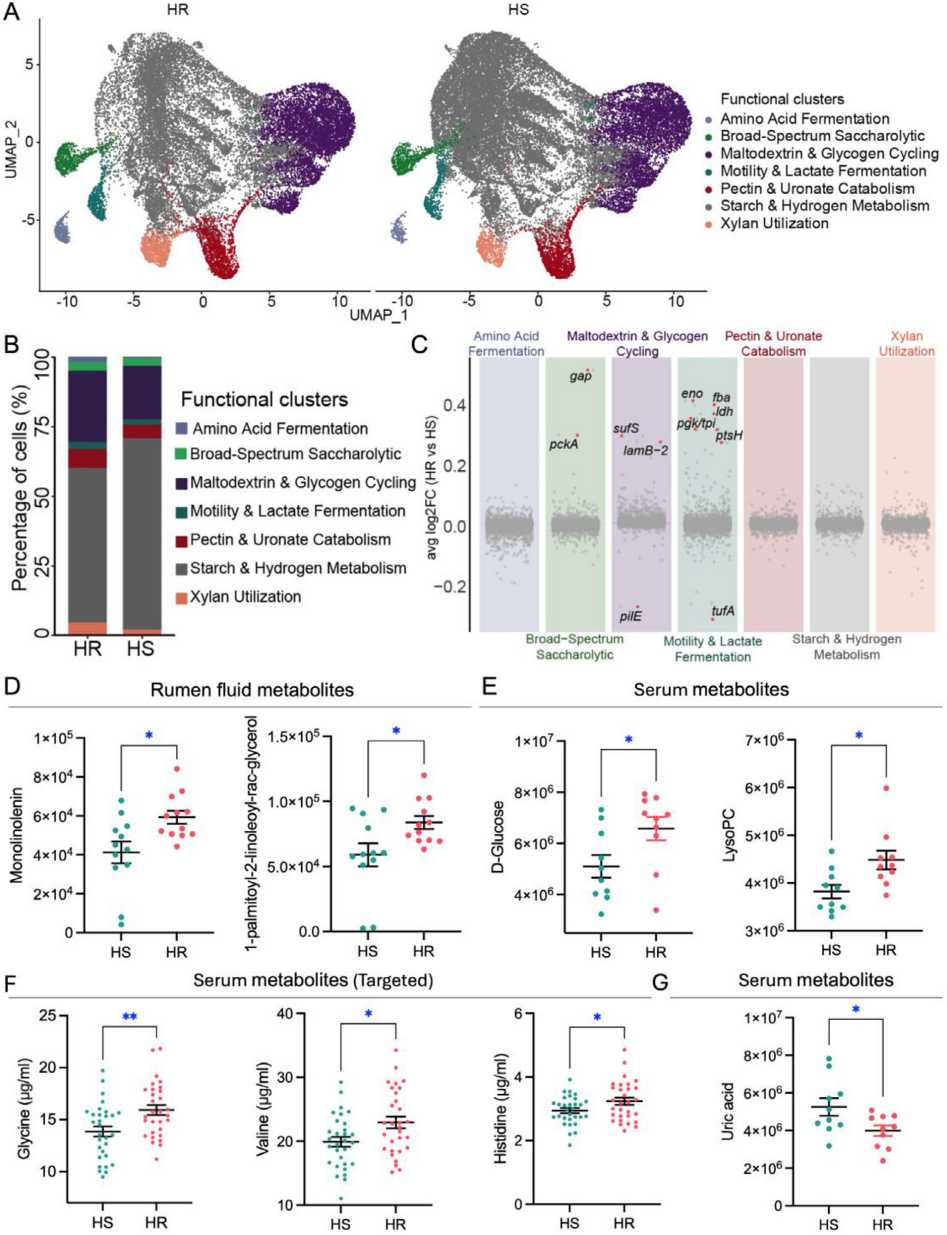
Functional rewiring of specific rumen clusters orchestrates host systemic metabolic resilience. (A) UMAP projection of all captured microbial single cells between Heat‐Resistant (HR) and Heat‐Sensitive (HS) hosts. (B) Bar charts detailing the relative proportion of cells within each functional cluster across the HR and HS cohorts. (C) Differential expression analysis identifies cluster‐specific transcriptional activation, highlighting the upregulation of glycolytic genes in HR hosts. (D) Non‐targeted rumen metabolomics reveals an enrichment of diacylglycerol and mono‐linolenin in HR rumen fluid. (E) Non‐targeted serum metabolomics indicates a shift toward high‐energy homeostasis in HR hosts, characterized by increased levels of D‐glucose and LysoPC. (F) Targeted quantification of serum amino acids shows elevated concentrations of valine, histidine, and glycine in HR sheep. (G) Significant reduction of serum uric acid in HR hosts, contradicting the expected profile of hyper‐catabolic nitrogen excretion associated with severe tissue breakdown. Data are presented as mean ± SEM. Each dot represents one biological sample, ^*^ *p* < 0.05, ^**^ *p* < 0.01.

To define the microbial basis of host thermal resilience, we integrated single‐cell transcriptomics with multi‐compartment metabolomics. Despite the extreme selective pressure of heat stress, the rumen microbiome maintained high structural conservation between HR and HS phenotypes. Both alpha‐diversity indices (Figure S10) and the relative distributions of the seven major functional clusters (Figure 4A,B, Table S9) remained statistically invariant. While high‐resolution profiling detected minor taxonomic fluctuations, including the depletion of *UBA3637* and *CAMVHY01* in HR hosts (Figure S11), these isolated shifts may not fully account for the robust physiological divergence observed.

In contrast to this structural conservation, single‐cell profiling revealed a targeted metabolic activation within specific carbohydrate‐active clusters (Figure 4C, Table S10). While the Amino Acid Fermentation, Starch & Hydrogen Metabolism, Pectin & Uronate Catabolism, and Xylan Utilization clusters showed minimal transcriptional variation, energy‐harvesting clusters exhibited synchronized upregulation of glycolytic genes in HR hosts. Specifically, the Motility & Lactate Fermentation cluster transitioned from a basal translational state (enriched in *tufA* in HS hosts) to a glycolytic phenotype in HR hosts, characterized by peak expression of rate‐limiting enzymes including *fba* (aldolase), *eno* (enolase), *ldh* (lactate dehydrogenase), and *pgk*/*tpi*. A similar trend was observed in the Broad‐Spectrum Saccharolytic and Maltodextrin clusters, where HR hosts upregulated genes for gluconeogenic precursors (*pckA*, *gap*) and high‐affinity sugar transport (*lamB‐2*), whereas HS hosts retained a structural adhesion profile (high *pilE*). These data indicate a localized transcriptional mobilization toward enhanced glycolytic capacity under stress.

Parallel metabolomic profiling revealed distinct alterations in both the local and systemic host metabolome. Elevated microbial activity in the rumen fluid of HR sheep was associated with increased lipid signaling molecules (monolinolenin, 1‐diacylglycerol‐2‐linoleoyl‐rac‐glycerol) (Figure 4D, Table S11). Systemically, the serum metabolome of HR animals was enriched for D‐glucose, LysoPC, 3‐Indoxyl sulphate and 3,4‐Dihydroxyphenylpropionic acid, indicating a distinct systemic energy and metabolic state (Figure 4E and Figure S12A, Table S12). Furthermore, we detected elevated levels of essential amino acids (valine, histidine, lysine, phenylalanine, and isoleucine) and glycine in HR serum (Figure 4F and Figure S12A, Tables S12 and S13).

A plausible physiological alternative for this increase in serum amino acids is that severe heat stress may induce skeletal muscle catabolism, leading to the release of free amino acids into the bloodstream [28, 29]. To evaluate this possibility, we evaluated relevant systemic catabolic and nitrogen excretion biomarkers. Notably, serum levels of 1‐methyl‐L‐histidine (a commonly used indicator of myofibrillar degradation), creatinine, and creatine showed no significant differences between the HR and HS cohorts (Figure S12B, Table S12). Moreover, serum urea remained stable (Figure S12B, Table S12), while uric acid was significantly lower in the HR group (Figure 4G, Table S12), which does not support an increased nitrogen catabolism profile in HR sheep. Furthermore, although branched‐chain amino acids increased (e.g., valine and isoleucine), their downstream degradation product, 3‐methyl‐2‐oxovaleric acid, along with 5‐Oxo‐D‐prolyl‐L‐valine, did not differ between groups (Figure S12B, Table S12). The stability of these catabolic markers suggests that the amino acid enrichment in HR sheep is unlikely to be primarily explained by greater muscle breakdown relative to HS sheep.

To explore this host‐microbiome crosstalk, we performed an integrative multi‐omics correlation analysis (Figure S13). In HR sheep, ruminal monolinolenin levels scaled positively with the microbial gluconeogenic gene *pckA* (Figure S13A). Systemically, microbial *gap* expression tightly tracked serum L‐phenylalanine exclusively within the HR cohort. Notably, this cross‐kingdom metabolic synchrony collapsed in HS animals, where *gap* transcription instead showed an inverse relationship with serum D‐Glucose (Figure S13C). While these cross‐omics correlations are observational and do not establish absolute causality, we hypothesize that they reflect a potential “nutritional sparing” effect. In this model, the observed enrichment of essential amino acids in HR sheep is closely associated with a state of physiological energy sufficiency, highlighting how microbiome‐derived energy subsidies may help buffer the host against environmental energy deficits.

### 2.5 Single‐Cell Resolution of *Anaerovibrio Lipolyticus* Reveals a Lineage‐Specific Metabolic Shift Mediating Host Thermotolerance

**FIGURE 5:**
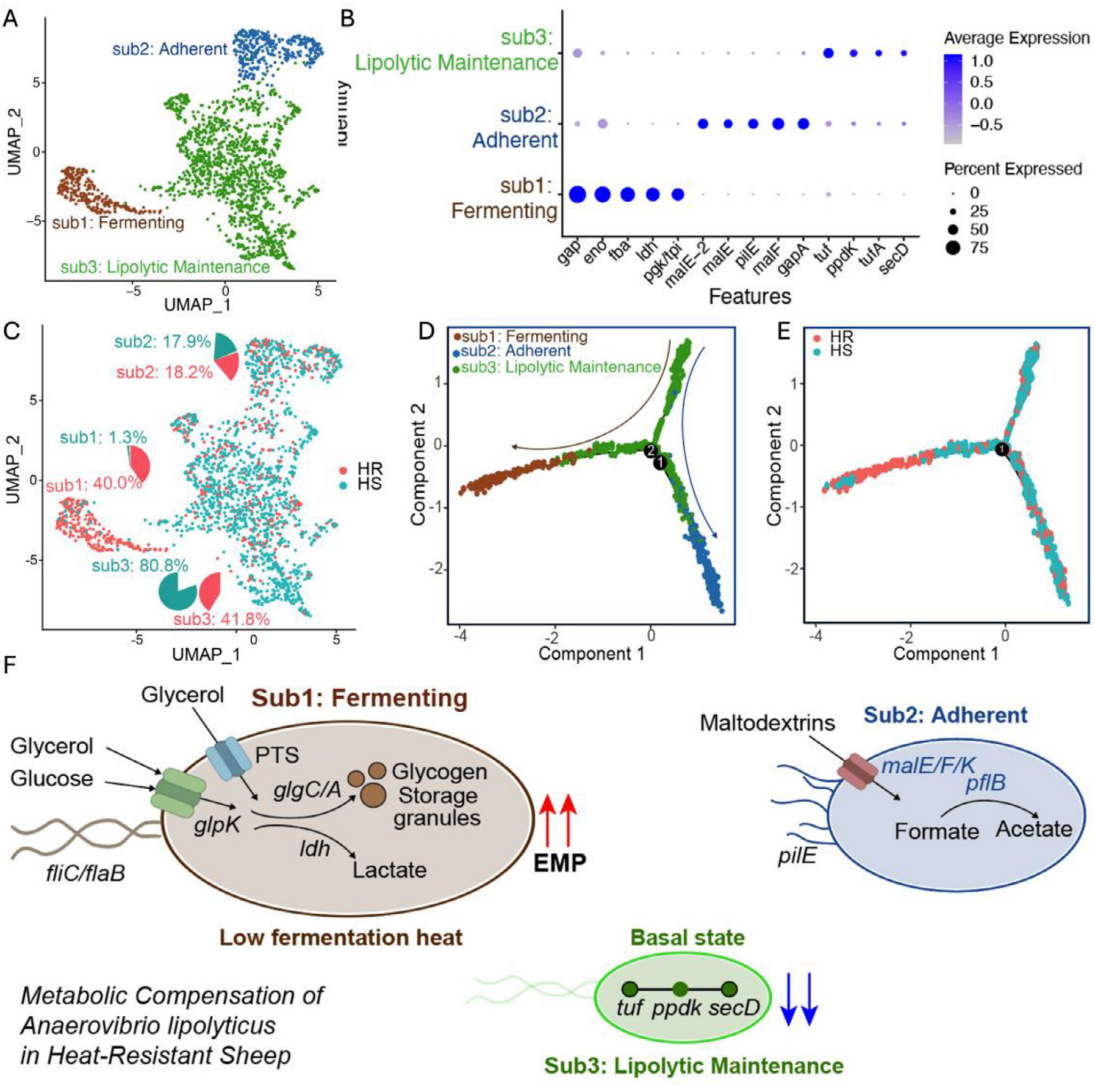
Single‐cell transcriptomic heterogeneity of *Anaerovibrio lipolyticus* associated with host thermotolerance. (A) UMAP embedding identifies three distinct transcriptional states within the *Anaerovibrio lipolyticus* population. (B) Dot plot displaying the expression of top marker genes defining each cluster. (C) Comparison of cluster abundance between groups. (D) Monocle2 pseudotime analysis inferring a transcriptional trajectory, with Sub3 set as the root and Sub1 as the terminal state. (E) Density of cells along pseudotime. (F) Proposed model of metabolic compensation.

To elucidate the cellular basis of this metabolic activation, we focused on *Anaerovibrio lipolyticus*, a dominant member of the Motility & Lactate Fermentation cluster. Unsupervised clustering of single‐cell transcriptomes identified three distinct transcriptional states of *Anaerovibrio lipolyticus* cells (Figure 5A), each characterized by specific gene modules (Figure 5B, Table S14). Sub‐cluster 3 (Sub3) represents a “Basal/Lipolytic maintenance” state, defined by the expression of ribosomal proteins (*rps*) consistent with biomass accumulation. Sub‐cluster 2 (Sub2) corresponds to an “Adherent” state, enriched for type IV pili (*pilE*) and maltose transporters (*malE*, *malF*) to facilitate substrate attachment. Sub‐cluster 1 (Sub1) constitutes a specialized “Fermenting” state, distinguished by the upregulation of central glycolytic enzymes, including glyceraldehyde‐3‐phosphate dehydrogenase (*gap*), enolase (*eno*), fructose‐bisphosphate aldolase (*fba*), and lactate dehydrogenase (*ldh*).

Comparative quantification revealed a distinct metabolic transition associated with host thermotolerance (Figure 5C). In HS hosts, the population was dominated by the Sub3 translation state (80.8%). Conversely, HR hosts exhibited a shift in population structure, with the abundance of Sub3 decreasing to 42%, while the glycolytic Sub1 lineage underwent a substantial expansion to comprise 40.0% of the population, compared to a mere 1.3% in HS hosts.

Given its basal homeostatic profile, Sub3 was designated as the root of the pseudotime trajectory (Figure S14), which reconstructed a transcriptional progression linking these distinct states (Figure 5D,E). In HS microbiomes, cells were predominantly retained at the trajectory origin (Sub3), consistent with the maintenance of translational machinery. Conversely, the HR microbiome exhibited a shift along the trajectory toward the Sub1 terminus. This distribution suggests that thermotolerance is associated with a transcriptional transition that prioritizes energy generation over biomass accumulation.

Based on these trajectory dynamics, we propose a model of metabolic compensation (Figure 5F). The induction of the *gap‐eno‐fba‐ldh* module at the pseudotime terminus characterizes the Sub1 phenotype. In contrast to the translation‐driven Sub3 population, the Sub1 lineage is transcriptionally configured to enhance flux through the Embden‐Meyerhof‐Parnas (EMP) pathway. This reprogramming facilitates the conversion of substrates into lactate and downstream energy precursors, offering a compensatory fuel source to offset host energy deficits associated with heat stress.

## 3 Discussion

Elucidating the transcriptional programs that govern host‐microbiome co‐adaptation has long been impeded by the fundamental technical trade‐off of achieving efficient microbial cell wall permeabilization without compromising labile RNA. Conventional single‐cell in situ reverse transcription protocols frequently introduce systematic capture biases, preferentially profiling fragile Gram‐negative bacteria while underrepresenting structurally robust populations, such as thick‐walled Firmicutes and methanogenic archaea [7, 30]. Accordingly, foundational efforts like the previous bovine rumen single‐cell atlas primarily provided a baseline view, constrained by these lysis limitations and the absence of environmental perturbations [8]. Here, we overcame this barrier by developing a time‐resolved enzymatic permeabilization strategy and applying it to a sheep model of thermotolerance divergence. This optimized approach successfully breached the complex envelopes of lysis‐resistant taxa, most notably the pseudo‐murein of the recalcitrant archaeal family *Methanomethylophilaceae*, while preserving the integrity of low‐abundance mRNA. Although the resulting capture rate (averaging 125 genes per cell) diverges from typical eukaryotic metrics, it aligns precisely with state‐of‐the‐art microbial single‐cell platforms (e.g., PETRI‐seq and microSPLiT) [15, 16]. Rather than a technical deficit, this reflects the intrinsically low mRNA content and rapid transcript turnover characteristic of prokaryotes [31, 32]. Furthermore, resolving distinct metabolic states within this highly complex ecosystem is not dependent on deep individual cellular coverage. Instead, it is driven by the statistical power of aggregating core co‐expression modules across a massive cellular cohort (n > 60 000), effectively buffering single‐cell dropout noise [33]. By moving beyond community‐averaged perspectives, this unbiased profiling allowed us to illuminate the ecosystem's “functional dark matter.” Most importantly, our findings support an association between microbial transcriptional reprogramming and host rumen resilience under heat stress, elevating transcriptional plasticity as a critical dynamic parallel to taxonomic shifts. Ultimately, this protocol provides a generalizable, culture‐independent framework for the in situ functional dissection of recalcitrant microbiomes across diverse agricultural, environmental, and clinical settings.

Our single‐cell framework suggests the rumen microbiome not merely as a random assemblage of taxa, but potentially as a highly organized, spatially partitioned digestive consortium. We observed a transcriptional correlation between microbial lifestyle and metabolic specialization, pointing toward a sophisticated division of labor. We hypothesize that an inferred spatial proximity between adherent, fibrolytic populations (e.g., *Cryptobacteroides*) and planktonic, fermentative satellites (e.g., *Anaerovibrio*) would facilitate an efficient cross‐feeding network. Ecologically, such proposed spatial segregation could enhance thermodynamic efficiency by accelerating interspecies substrate transfer and mitigating local product inhibition [31, 34]. Furthermore, the stable intracellular pH signatures in adherent cells imply that biofilm‐associated lifestyles may create buffered microniches, potentially shielding delicate primary degraders from acidic fermentation byproducts. Ultimately, this inferred spatial‐metabolic structuring is likely required for the continuous degradation of recalcitrant fibers within a highly dynamic environment [35].

Another paramount implication of this study is that host thermal resilience is associated with targeted transcriptional reprogramming within pre‐existing microbial populations, acting independently of large‐scale taxonomic turnover. This aligns with the paradigm of functional redundancy, wherein microbiome metabolic output remains robust despite compositional stasis under stress [36, 37]. The marked transition of the *Anaerovibrio lipolyticus* Sub1 lineage from a basal translational state to a highly glycolytic phenotype (characterized by robust *ldh*, *eno*, and *fba* expression) epitomizes phenotypic plasticity as a superior, rapid‐response strategy compared to the slower dynamics of ecological succession [16]. Notably, these microbial metabolic changes appear to be associated with host ingestive behavior and energy extraction. While the HR cohort maintained higher average daily feed intake, providing an increased substrate pool, the observed transcriptional shifts suggest that the microbiome does not merely respond passively to increased substrate. Instead, the activation of glycolytic flux facilitates the conversion of available substrates into terminal fermentation products, particularly lactate and propionate [38]. This potential combined effect suggests that the host's sustained intake is channeled into primary gluconeogenic precursors, which support hepatic glucose synthesis and actively compensate for the environmental energy deficit [39, 40]. This prioritization of energy‐generating pathways allows the rumen microbiome to serve as a metabolic buffer, creating a physiological context that reduces the host's need to mobilize its endogenous reserves for energy homeostasis.

Building on these cross‐omics correlations and the stability of systemic catabolic biomarkers, we propose a plausible working model in which microbial energy supply may contribute to a systemic “nutritional sparing” effect. While severe thermal stress typically induces skeletal muscle catabolism and a consequent release of free amino acids [41, 42], this explanation is not strongly supported by the metabolic signature observed in the HR cohort. Muscle breakdown is generally associated with severe physiological strain and systemic energy deficits [43, 44]. As detailed by the absence of elevated creatinine, 1‐methyl‐L‐histidine, and nitrogenous waste products in our results, the amino acid enrichment in our study was exclusive to the HR animals that successfully maintained core homeostasis, rather than the highly stressed HS group. Furthermore, their concurrent elevation of circulating D‐glucose and lipids points to a state of robust energy sufficiency, a physiological condition that actively suppresses muscle mobilization [45]. Consequently, this proposed microbial‐driven “nutritional sparing” effect likely helps safeguard host tissues from pathological breakdown and attenuates systemic strain, highlighting its potential role in supporting host thermal resilience.

In conclusion, this high‐resolution single‐cell atlas transitions our understanding of the rumen microbiome from a static taxonomic inventory to a dynamic map of cellular behaviors. By resolving transcriptional heterogeneity within a single species, we can link micro‐scale cellular states directly to macro‐scale host phenotypes, suggesting that future strategies for improving livestock resilience might focus on modulating endogenous transcriptional programs rather than solely relying on exogenous probiotic introductions. However, we acknowledge certain biological, technical, and computational limitations in the current framework. Biologically, our natural summer‐stress cohort lacks a thermoneutral control to define an absolute baseline for generalized heat stress. Instead, this model is explicitly designed to resolve intraspecific thermal resilience, identifying the compensatory mechanisms that allow specific individuals to maintain homeostasis under identical environmental pressures. While this approach successfully captures targeted metabolic adaptations (such as the glycolytic shift in *Anaerovibrio lipolyticus*), these findings remain fundamentally observational and are restricted to a single ovine model (Hu sheep). Transitioning from systemic correlation to definitive causality demands rigorous mechanistic validation. Future work must prioritize the targeted isolation of key rumen anaerobes for Cas9‐mediated genetic engineering [46, 47], allowing their functional impact to be directly evaluated via emerging rumen epithelial organoids or controlled in vivo inoculations [48, 49]. Technically, although our optimized pipeline successfully permeabilized recalcitrant archaeal lineages, their low ecological abundance resulted in sparse cellular recovery across the 21‐sample cohort. This sparsity precluded robust differential expression profiling of methanogenic pathways. To deeply interrogate these rare taxa, future protocols must integrate immediate cellular fixation with FACS‐based pre‐enrichment (e.g., via F420 autofluorescence) prior to single‐cell encapsulation [50, 51, 52]. Computationally, the use of standard pipelines such as STARsolo is constrained by the presence of highly conserved sequences in microbial genomes, leading to ambiguous read mapping and a trade‐off between read utilization and assignment specificity when restricting quantification to uniquely assigned reads. Consistent with established analytical frameworks [15, 53], our multidimensional clustering relies on thousands of highly variable genes, rendering the definition of cellular states inherently robust to individual transcript variance [54]. Concurrently, developing microbiome‐specific smRandom‐seq pipelines equipped with advanced probabilistic read‐assignment models will be crucial to fully resolve these transcriptional nuances. Ultimately, the methodology established here provides a robust and broadly applicable tool for dissecting functional dynamics within complex microbiomes. Expanding upon this foundation, future cross‐species integrations of ovine and bovine single‐cell atlases will be instrumental in elucidating conserved, microbiome‐mediated adaptation mechanisms across ruminant livestock.

## 4 Materials and Methods

### 4.1 Ethics, Animals, and Experimental Design

All experimental procedures were approved by the Animal Ethics and Welfare Committee of Guangxi University (Approval No. GXU‐2025‐144). The study was conducted at the Anxing Hu Sheep Core Breeding Farm (Guangxi, China) during the summer season (June to September), with a Temperature‐Humidity Index (THI) of 78–89.

An initial cohort of 1813 healthy, multiparous, non‐pregnant Hu sheep ewes (parity 2) was recruited for phenotypic screening. To ensure baseline uniformity, all individuals met strict inclusion criteria: a target body weight of 45.0–55.0 kg and a body condition score (BCS) of 2.75–3.25 (5‐point scale). Animals were maintained under standard commercial management and group‐fed a pelleted total mixed ration (TMR). Detailed dietary ingredients and analyzed chemical composition (100% dry matter basis) are provided in Tables S1 and S2. The herd had continuous ad libitum access to feed and fresh water, with daily intake visually monitored by farm staff to ensure constant feed availability. To evaluate feeding behavior under heat stress, average daily feed intake was recorded for 60 consecutive days. The total TMR offered and daily refusals (orts) per pen were weighed each morning. Individual ADFI was then determined by dividing the net pen‐level feed consumption by the number of animals.

### 4.2 Quantification of Physiological Indices

To assess thermotolerance, physiological parameters—respiratory rate (RR), rectal temperature (RT), and heart rate—were measured during the peak diurnal heat window (13:30–16:30). Measurements were strictly conditional: they were conducted only on days when the real‐time ambient Temperature‐Humidity Index (THI) met the threshold for moderate heat stress, and were suspended during unseasonal cooling or extreme heat events. To minimize handling stress, operations were performed concurrently by six trained researchers divided into two groups while animals were resting.

RR was visually quantified via flank undulations for 1 min. To eliminate observer bias, counts were recorded synchronously by three independent observers, and the mean was calculated. RT was measured using a sterilized digital thermometer inserted 4–5 cm into the rectum. Heart rate was assessed via auscultation at the third–5th intercostal space for 1 min. For both RT and heart rate, the final value for each animal was derived from the average of three consecutive measurements.

### 4.3 Multi‐Stage Phenotypic Screening Strategy

To isolate individuals with distinct and stable thermotolerant phenotypes and to effectively control for environmental variance, a longitudinal, three‐stage screening strategy was implemented from June to September [55] (as illustrated in Figure S2):

1. Baseline screening: The initial cohort of 1813 multiparous Hu sheep underwent primary evaluation based on RR and RT, identifying a candidate pool of 800 individuals.
2. Iterative stability filtering: To filter out transient physiological responses to month‐to‐month microclimate shifts, the candidates were subjected to three subsequent rounds of physiological evaluations, each separated by a 1‐week stabilization interval. This iterative culling narrowed the population sequentially from 800 to 300, and then to 100 individuals, retaining only those exhibiting consistent physiological responses.
3. Divergent selection: A comprehensive evaluation integrating heart rate with RR and RT was applied to the remaining 100 candidates. The 60 animals demonstrating the most consistent and divergent phenotypes across all testing rounds (30 HR and 30 HS) were ultimately selected for downstream biological sampling.

### 4.4 Statistical Classification and Calculation of Thermotolerance Indices

Phenotypic classification was mathematically defined using Principal Component Analysis (PCA) coupled with fuzzy membership functions to derive a comprehensive thermotolerance index (RW). Physiological responses to heat stress (RR, RT, and heart rate) exhibit substantial multicollinearity. To integrate these multi‐dimensional parameters into a single quantitative metric, we employed a combined PCA and fuzzy mathematical model—a well‐established statistical paradigm for evaluating multi‐trait adaptability [56, 57]. PCA was first utilized to eliminate multicollinearity and extract principal components representing overall thermal strain. Subsequently, the membership value *R(Di)* for the *i*‐th principal component was calculated as:

$$R\left(Di\right)=\frac{Di-Dmin}{Dmax-Dmin}\;\left(i=1,2,\text{…},n\right)$$

where *Di* represents the value of the *i*‐th principal component, and *Dmax* and *Dmin* denote its maximum and minimum values, respectively. Subsequently, the final comprehensive evaluation value (*RW*) was calculated by weighting the membership values according to their component contributions:

$$RW=\sum_{i=1}^{n}Wi\times R\left(Di\right)$$

where *Wi* represents the importance weight of the *i*‐th principal component, derived from its contribution rate (*Ki*) relative to the cumulative contribution of all selected components. Individuals with higher *RW* values were classified as HS, while those with lower values were classified as HR.

### 4.5 Biological Sampling Collection

At the conclusion of the phenotypic screening in mid‐September, biological sampling was conducted on the final cohort of 60 selected sheep. To minimize circadian and post‐prandial metabolic variations, all procedures were performed strictly before the morning feeding. Specifically, animals underwent a 24 h fast and a 2 h water deprivation prior to sampling. All biological samples were collected consecutively within a tightly controlled 3 h window (09:00–12:00) by two synchronous, trained research teams. For each individual, venous blood was drawn before rumen fluid extraction to limit stress‐induced physiological fluctuations. Blood was collected via jugular puncture into 10 mL non‐anticoagulant vacuum tubes and allowed to clot at room‐temperature for 30 min. After centrifugation at 3000 × g for 15 min at 4°C, the supernatant serum was immediately aliquoted and stored at ‐80°C. Subsequently, rumen fluid was obtained using an oral esophageal tube. The initial 150 mL of aspirate was discarded to prevent saliva contamination, and the remaining fluid was filtered through two layers of cheesecloth, flash‐frozen in liquid nitrogen, and stored at ‐80°C for downstream analysis. From this main cohort, 24 animals (12 HR and 12 HS) were selected for untargeted metabolomic profiling. Concurrently, a representative subset of 21 matched samples (11 HR and 10 HS) was allocated for smRandom‐seq.

### 4.6 Optimization of Cell Wall Lysis for Unbiased Archaeal Capture

According to the protocol previously established by the laboratory [8, 17, 18, 58], rumen fluid samples were fixed in 4% paraformaldehyde (PFA) with overnight agitation at 4°C. Following fixation, impurities were removed via centrifugation (500 × g, 5 min) and 20 µm filtration. The cell suspension was calibrated to 3 × 10^6^ cells and treated with 0.06% Tween‐20 for 5 min at 4°C. Subsequently, cells underwent controlled digestion using a tailored enzymatic mix (100 mm Tris [pH 8.0], 50 mm EDTA, 0.25 U/µL RNase Inhibitor, 2.5 mg/mL lysozyme, 12.5 ng/µL lysostaphin) at 37°C. The optimal digestion duration of 25 min was determined through time‐course evaluations (15, 25, and 35 min). Briefly, a pure culture of *Methanobacterium ruminantium* (BMZ063901) and the complex rumen microbiome were subjected to these enzymatic treatments. For visual assessment, treated cells were stained with DAPI and examined via combined brightfield and fluorescence microscopy to confirm intracellular nucleic acid accessibility alongside morphological preservation. Concurrently, qPCR cycle threshold (Ct) values were measured to quantify target DNA release and monitor potential cell leakage. Together, these assays confirmed that a 25 min treatment effectively permeabilizes archaeal pseudo‐murein walls while preventing the over‐digestion and lysis of more fragile bacterial taxa. Following this strictly defined 25 min digestion, the reaction was immediately quenched by resuspension in ice‐cold PBS‐RI (1× PBS supplemented with 0.05 U/µL RNase inhibitor). Finally, cells were washed and resuspended in 35.5 µL of ice‐cold PBS‐RI at a final density of 1 × 10^6^ cells for downstream in situ reactions.

### 4.7 In Situ Reverse Transcription and dA‐Tailing

In situ reactions were performed using the VITApilote‐PFT1200 kit (M20 Genomics, R20114124). For reverse transcription (RT), an 80 µL reaction mixture was added to the cell suspension, comprising 16 µL of 5× RT buffer, 12 × 1.5 µL of random primers (10 µm; Table S3), 3.5 µL of dNTPs (10 mm), 3.5 µL of RNase inhibitor, and 3.5 µL of Reverse Transcriptase (50 U/µL). Thermal cycling was conducted using a progressive annealing ramp (12 cycles increasing from 8°C to 42°C), followed by incubation at 42°C for 30 min and 50°C for 30 min. The reaction was terminated by the addition of 1 µL 50 mM EDTA. Cells were subsequently washed five times with ice‐cold PBS‐T (0.05% Tween‐20 in PBS) and resuspended in 39.5 µL of PBS‐T. For in situ dA‐tailing, cells were resuspended in a reaction solution containing 5 µL 10× TdT buffer, 5 µL 2.5 mm CoCl_2_, 0.5 µL 100 mm dATP, and 0.5 µL TdT enzyme. The mixture was incubated at 37°C for 30 min, after which the cells were washed three times and resuspended in 100 µL of PBS‐T for downstream processing.

### 4.8 Single‐Microbe High‐Throughput Droplet Encapsulation

High‐throughput droplet encapsulation was performed using the VITAcruizer DP400 microfluidic platform (M20 Genomics). Prior to encapsulation, bacterial cell density was quantified via microscopy and adjusted to 400 cells/µL in a 30% density gradient solution to prevent sedimentation. The cell suspension, alongside barcoded hydrogel beads and a 4× DNA extension mix, was loaded into the designated inlets of the microfluidic chip. Following generation, the droplets were subjected to a programmed thermal incubation protocol (37°C for 60 min, 50°C for 30 min, 60°C for 30 min, and 75°C for 20 min) to facilitate simultaneous cell lysis and barcode extension.

### 4.9 CDNA Enrichment and Library Construction

Droplets were demulsified using PFO buffer to isolate the aqueous phase. cDNA was purified using DNA cleanup magnetic beads (Beckman, A63882), and the optimal PCR cycle number for enrichment was determined via qPCR (Table S3). Enriched cDNA was purified and quantified using a Qubit 3.0 Fluorometer (Thermo Fisher), with fragment size distribution assessed on a Qsep100 DNA Fragment Analyzer (Bioptic). Sequencing libraries were constructed using the VAHTS Universal Pro DNA Library Prep Kit for Illumina V3 (Vazyme, ND607‐02). Qualified cDNA underwent simultaneous end‐repair and dA‐tailing at 20°C for 30 min, followed by enzyme inactivation at 65°C for 30 min. Subsequently, adapter ligation was performed at 20°C for 30 min. Post‐ligation libraries were purified and size‐selected using magnetic beads. Final libraries were amplified, purified, and sequenced on an Illumina NovaSeq 6000 platform (PE150 mode).

### 4.10 Reads Preprocessing and Taxonomic Annotation

Raw paired‐end FASTQ files generated from microbial single‐cell RNA‐seq libraries were processed using a custom parsing pipeline based on the smRandom‐seq workflow (https://github.com/WangycLab/smRandom‐seq‐protocol) [17, 18]. The pipeline comprised three major steps: read preprocessing, taxonomic annotation, and construction of cell‐by‐gene count matrices. During read preprocessing, sequencing reads were parsed according to the expected library structure to extract 20‐bp cell barcodes (CBs), 8‐bp unique molecular identifiers (UMIs), and the corresponding cDNA sequences. For taxonomic annotation and reference‐guided quantification, quality‐controlled reads were classified using Kraken2 (v2.1.3) against a curated microbial reference derived from the Sheep Rumen Genome Catalogue v1.0 (EMBL‐EBI MGnify). To prevent read‐mapping artifacts driven by conserved sequence homologies, transient environmental and feed‐borne taxa (e.g., *Nanosyncoccus*, *RF16*, and *UBA2834*) were explicitly excluded from the reference index prior to alignment [59, 60]. Retaining these non‐indigenous genomes allows them to act as erroneous mapping sinks for transcriptomic reads, which artificially inflates their abundance and distorts the quantitative profile of the resident microbiota [61, 62]. The customized Kraken2 database was generated by filtering the corresponding genome FASTA files and seqid‐to‐taxid mappings without reassigning taxonomy identifiers. Finally, species supported by a minimum of 50 reads per sample were retained to construct sample‐specific reference genomes for downstream quantification.

### 4.11 Reads Alignment and Count Matrix Construction

Based on Kraken2 species assignments, sample‐specific reference genomes were constructed and indexed using STAR (v2.7.10b). Reads were aligned and quantified using STARsolo in CB_UMI_Simple mode. Gene‐level quantification was performed in GeneFull mode with reverse‐strand specificity, and cell calling was conducted using the CellRanger2.2 algorithm implemented in STARsolo. Multi‐mapping reads were retained during alignment under default STAR settings and annotated with NH tags (–outSAMattributes NH HI AS CR UR GX GN), but were handled conservatively during gene‐level quantification (GeneFull mode without explicit multi‐mapper reassignment). Only uniquely assigned reads contributed to gene expression estimates. For downstream analyses, cells were ranked by total UMI counts within each sample, and the top 5000 cells per sample were retained to reduce sparsity and ensure comparable cell numbers across samples.

### 4.12 Data Integration and Quality Control Using Seurat

Count matrices from all samples were imported into R (v4.2.2) and analyzed using Seurat (v4.3.0). For each sample, expression matrices were loaded in 10Í format and converted into Seurat objects with a minimum threshold of three cells per gene and three detected genes per cell. Kraken2‐based taxonomic annotations at the species and genus levels were integrated into Seurat metadata by matching cell barcodes. Seurat objects from all samples were merged into a single combined dataset. Cells with total UMI counts ≤ 50 or ≥ 10 000 were removed. In addition, cells with total UMI counts outside the 20th to 90th percentile range within each sample were excluded. The remaining cells were retained for downstream analyses.

### 4.13 Species‐ and Genus‐Level Composition Analyses

Species‐ and genus‐level composition profiles were computed based on Seurat metadata. Cell‐level taxonomic annotations were used to quantify the distribution of detected taxa across samples and experimental groups. Species and genera were ranked by the number of associated cells, and the top 20 most abundant taxa were summarized at the global, sample, and group levels. For each sample, relative abundances were calculated as the fraction of cells assigned to each taxon. Basic quality metrics, including the number of detected genes and total UMI counts per cell, were summarized and compared across samples and groups.

### 4.14 Between‐group Comparison of Microbial Composition

Comparative analyses between HS and HR groups were performed using species‐ and genus‐level cell composition profiles. To characterize within‐sample diversity, alpha diversity indices (including Observed species, Shannon, and Simpson) were calculated. Statistical significance between the HR and HS groups was determined using the Wilcoxon rank‐sum test. Effect sizes were quantified as log_2_ fold changes between group means. Taxa detected in both groups and showing *p* values < 0.05 were considered group‐associated.

### 4.15 Gene Annotation, Dimensionality Reduction, and Unsupervised Clustering

To harmonize annotations across genomes, gene identifiers were mapped to symbols based on the reference GTF, with KEGG Orthology (KO) identifiers assigned utilizing the Kyoto Encyclopedia of Genes and Genomes (KEGG) database [63, 64]. Expression counts corresponding to identical gene symbols were aggregated, while unannotated genes retained their original identifiers. The merged count matrix was normalized using SCTransform, with total UMI counts (nCount_RNA) regressed out and 10 000 highly variable genes identified. Following SCTransform normalization, we rigorously evaluated the necessity of algorithmic batch correction. Silhouette analysis confirmed that cells across the 21 biological replicates were inherently well‐mixed (negative sample‐level silhouette score), indicating effective normalization of technical variance. Crucially, applying batch correction algorithms (e.g., Harmony) introduced detrimental biological over‐correction, artificially eroding the genuine phenotypic divergence between the HR and HS cohorts. Therefore, to preserve true host‐driven biological heterogeneity, the unintegrated SCTransform dataset was retained. Principal component analysis (PCA) was performed, and the top principal components were utilized to construct a shared nearest‐neighbor (SNN) graph and derive two‐dimensional UMAP embeddings.

For unsupervised clustering, the Louvain algorithm was applied. To ensure the robustness of our cell population definitions, we assessed cluster stability using a clustering tree analysis (*clustree* R package) across a resolution gradient from 0.2 to 0.6 (Figure S6). The visualization confirmed highly stable topological lineages. To prevent a single low‐resolution parameter from masking subtle transcriptomic heterogeneity, we employed an over‐clustering and functional merging strategy. We established a high‐resolution baseline at a resolution of 0.4, yielding 29 distinct sub‐clusters (clusters 0–28). These sub‐clusters were subsequently evaluated for shared marker expression and biological pathways. Sub‐clusters exhibiting highly similar transcriptomic profiles and shared core functions were merged, resulting in the final seven distinct functional macro‐clusters. This systematic approach ensures the defined clusters represent mathematically stable and true biological states shared across all 21 replicates, rather than algorithmic or sample‐specific artifacts. Finally, differentially expressed genes between the HR and HS cohorts within these validated functional clusters were defined by an absolute log_2_ fold change > 0.25 and an adjusted *p*‐value (FDR) < 0.05.

### 4.16 Differential Gene Expression Analysis

Differential gene expression analysis was performed to identify marker genes associated with each cluster type. Cells were grouped according to manually curated cluster type annotations derived from Seurat clustering results. Differential expression testing was conducted using Seurat's FindAllMarkers function with the Wilcoxon rank‐sum test. Genes were required to be detected in at least 1% of cells within a cluster (min.pct = 0.01) and to exhibit an absolute log_2_ fold change greater than 0.1. Only genes showing higher expression in a given cluster type relative to all other clusters were retained (only.pos = TRUE). For each cluster type, marker genes were ranked by average log_2_ fold change, and the top 10 genes were selected for visualization. A second set of top 10 marker genes, excluding MGYG‐prefixed identifiers, was generated to reduce redundancy from uncharacterized genome‐derived gene names. Furthermore, to identify differentially expressed genes between the HR and HS groups within each functional cluster, differential testing was performed at the single‐cell level using the Wilcoxon rank‐sum test. Statistical significance was strictly defined using thresholds of an absolute log_2_ fold change > 0.25 and a Benjamini‐Hochberg adjusted *p*‐value (FDR) < 0.05.

### 4.17 Species‐Specific Subclustering and Pseudotime Analysis

To resolve transcriptional heterogeneity within dominant microbial populations, species‐specific subclustering analyses were performed for *Cryptobacteroides sp900318445* and *Anaerovibrio lipolyticus*. Cells belonging to each species were analyzed independently using identical preprocessing, dimensionality reduction, and clustering parameters to minimize cross‐species effects. For each species, pseudotime‐associated transcriptional programs were characterized using Monocle2 (v2.26.0) [8, 65]. Cells with undefined pseudotime values were excluded prior to analysis. Genes were filtered to retain those detected in at least 5% of cells with non‐zero mean expression. Among these candidates, the top 100 genes with the highest empirical dispersion were selected as representative pseudotime‐associated genes. Expression dynamics were visualized using Monocle2 pseudotime heatmaps, and genes were grouped into four co‐expression modules based on hierarchical clustering of their pseudotime expression profiles. Module assignments were extracted from the heatmap row dendrogram for downstream interpretation.

### 4.18 Untargeted and Targeted Metabolomic Profiling of Rumen Fluid and Serum

To comprehensively map the metabolic landscape and quantify specific metabolic shifts, we employed a combined untargeted and targeted metabolomics approach. For global untargeted profiling of both rumen fluid and serum, metabolites were extracted using pre‐chilled organic solvents (e.g., methanol/acetonitrile). Following centrifugation, the supernatants were subjected to high‐resolution chromatographic separation coupled with mass spectrometry. Data acquisition was performed in both positive and negative electrospray ionization (ESI) modes to maximize metabolite coverage. Raw data processing, including peak alignment and picking, was conducted, followed by metabolite annotation against standard reference databases (e.g., HMDB and KEGG) to identify differential metabolic signatures.

For the absolute quantification of 22 specific amino acids in the serum, targeted metabolomics was executed utilizing an ultra‐performance liquid chromatography‐tandem mass spectrometry (UPLC‐MS/MS) system (ACQUITY UPLC I‐Class coupled to an Xevo TQ‐S Micro, Waters, USA) at Shanghai Personal Biotechnology Co., Ltd. (Shanghai, China). Briefly, 50 µL of serum was spiked with a mixture of isotope‐labeled internal standards to robustly correct for matrix effects and recovery loss. Following protein precipitation using pre‐chilled organic solvents, the supernatant was separated on an ACQUITY UPLC BEH Amide column (2.1 × 100 mm, 1.7 µm). Target analytes were detected in multiple reaction monitoring (MRM) mode via ESI. Absolute quantification was achieved using standard calibration curves exhibiting high linearity (R^2^ > 0.99). Data acquisition and targeted quantitative processing were performed utilizing MassLynx software (v4.1, Waters).

Statistical analyses and data visualizations were executed using R and GraphPad Prism 10 (v10.0.3). Routine quantitative comparisons between the HR and HS cohorts were assessed via two‐tailed unpaired Student's *t*‐tests. For multi‐omics integration, Spearman's rank correlations were employed to link microbial transcriptional activity with host metabolic phenotypes. Sample‐level pseudobulk gene expression was correlated against corresponding metabolite abundances—utilizing relative intensities and absolute concentrations for the untargeted and targeted platforms, respectively. To resolve state‐dependent metabolic architectures, these correlation matrices were computed independently within the HR (*n* = 11) and HS (*n* = 10) cohorts. Cross‐omics significance was determined using *P*‐values. Exact statistical tests for individual panels are annotated in the respective figure legends, with universal significance thresholds defined as *p* < 0.05 (^*^), *p* < 0.01 (^**^), and *p* < 0.001 (^***^).

## Author Contributions

S.Z., Y.W., and Z.X. designed the research. S.Z. and Z.X. performed the single‐cell experiments, S.Z., Q.J., J.M., Y.H., Y.W., and Z.X. analyzed the single‐cell data, J.Z., F.W., F.L., and Y.H. assisted with the data processing. S.Z. wrote the initial draft of the manuscript, S.Z., Q.J., Y.W., and Z.X. revised the manuscript. All authors read, reviewed, and approved the manuscript.

## Conflicts of Interest

The authors declare no conflicts of interest

## Supporting information




**Supporting File**: advs76152‐sup‐0001‐SuppMat.docx.

## Data Availability

The data that support the findings of this study are available from the corresponding author upon reasonable request.
