# Supplementary material for: A Single‐Cell Transcriptomic Atlas of the Ovine Rumen Microbiome Characterizes Lineage‐Specific Metabolic Shifts Associated with Host Heat Tolerance
Source: Adv Sci (Weinh). 2026 Jun 15:e76152. Online ahead of print. doi: 10.1002/advs.76152 (PMC13336951; doi:10.1002/advs.76152)
Supplement: Supplementary file 1 — Supporting File: advs76152‐sup‐0001‐SuppMat.docx. [file ADVS-9999-e76152-s001.docx]

**A Single-Cell Transcriptomic Atlas of the Ovine Rumen Microbiome Characterizes Lineage-Specific Metabolic Shifts Associated with Host Heat Tolerance**

Sanbao Zhang^1, #^, Qinyang Jiang^3, #^, Junjie Ma^1, #^, Jianwei Zou^3^, Fan Wang^3^, Feifei Lv^1^, Yanna Huang^3^, Yongcheng Wang^1, *^, Ziye Xu^1, 2, *^

^1^ Department of Laboratory Medicine of The First Affiliated Hospital & Liangzhu Laboratory, Zhejiang University School of Medicine, Hangzhou, China

^2^ Zhejiang Key Laboratory of Clinical In Vitro Diagnostic Techniques, Hangzhou, China

^3^ Guangxi Key Laboratory of Animal Breeding, Disease Control and Prevention, College of Animal Science and Technology, Guangxi University, Nanning, China

^#^ These authors contributed equally to this work.

* Corresponding Author: Ziye Xu (ziyexu@zju.edu.cn), Yongcheng Wang (yongcheng@zju.edu.cn)

**Supplementary Tables**

Table S1. Ingredient composition of the basal diet (dry matter basis, %).

Table S2. Analyzed nutritional composition of the basal diets (Dry matter basis, %).

Table S3. Primer and adapter sequences for single-microbe RNA sequencing.

Table S4. List of genera and species identified via single-microbe RNA sequencing.

Table S5. List of marker genes and corresponding functional terms for the 7 microbial functional clusters. Functional annotations and KO identifiers were assigned based on the Kyoto Encyclopedia of Genes and Genomes (KEGG) database.

Table S6. Taxonomic composition and relative abundance of the top 20 genera within each of the 7 functional clusters.

Table S7. Taxonomic composition and relative abundance of the top 20 species within each of the 7 functional clusters.

Table S8. List of marker genes and corresponding functional terms for *Cryptobacteroides sp900318445* sub-cluster. Functional annotations and KO identifiers were assigned based on the KEGG database.

Table S9. Comparison of microbial cell abundance within functional clusters between heat-resistant (HR) and heat-sensitive (HS) sheep.

Table S10. Differential expression analysis identifies cluster-specific transcriptional changes between heat-resistant (HR) and heat-sensitive (HS).

Table S11. Differential metabolites identified in the rumen fluid of heat-resistant (HR) and heat-sensitive (HS) sheep via untargeted metabolomics.

Table S12. Differential metabolites identified in the serum of heat-resistant (HR) and heat-sensitive (HS) sheep via untargeted metabolomics.

Table S13. Absolute quantification of differential serum amino acids (glycine, valine, and histidine) between heat-resistant (HR) and heat-sensitive (HS) sheep.

Table S14. List of marker genes and corresponding functional terms for *Anaerovibrio lipolyticus* sub-cluster. Functional annotations and KO identifiers were assigned based on the KEGG database.

**Supplementary Figures**

**
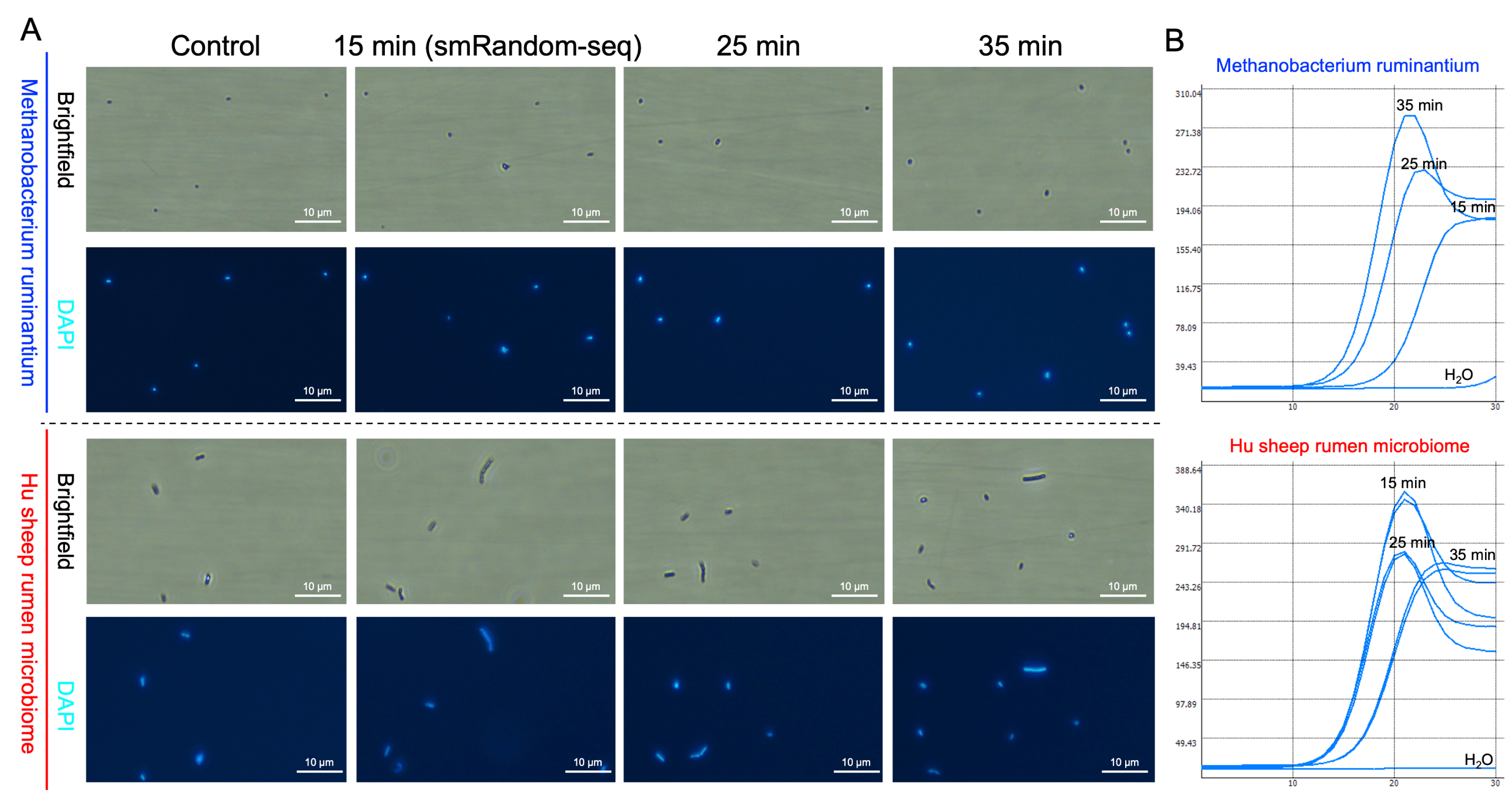
**

**Fig. S1** Empirical validation of the time-resolved enzymatic permeabilization window for *in situ* microbial single-cell profiling. (A) Representative brightfield and fluorescence (DAPI) micrographs of a recalcitrant archaeal isolate (*Methanobacterium ruminantium*, top panels) and the complex Hu sheep rumen microbiome (bottom panels) across an enzymatic digestion time-course (untreated, 15, 25, and 35 min). Scale bars, 10 µm. (B) Real-time quantitative PCR (qPCR) amplification curves used to assess nucleic acid release across digestion conditions. Ct values were determined using a fixed fluorescence threshold within the exponential phase of amplification curves.


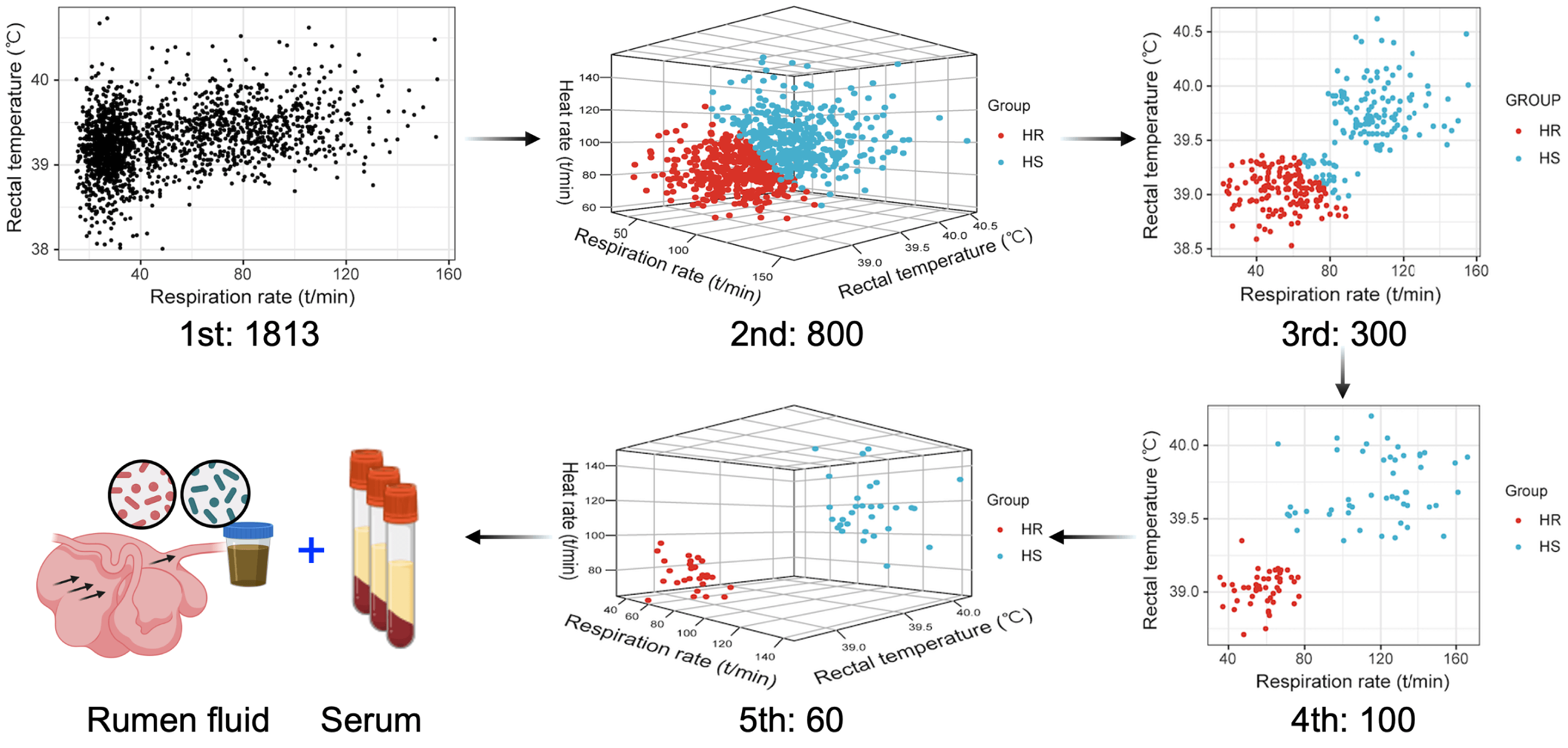


**Fig. S2** Schematic illustration of the multi-stage phenotypic screening strategy. The flowchart depicts the hierarchical selection process used to isolate heat-resistant (HR) and heat-sensitive (HS) Hu sheep from an initial population of 1,813 individuals. The cohort was sequentially refined through five stages (n = 1,813, 800, 300, 100, 60) based on key physiological indices: respiratory rate, rectal temperature, and heart rate. Scatter plots illustrate the progressive stratification, where red dots represent the HR group (characterized by lower physiological indices) and blue dots represent the HS group (characterized by higher physiological indices).

**Fig. S3** Physiological and feeding metrics (respiratory rate, rectal temperature, heart rate, and average daily feed intake) confirming significant phenotypic divergence between HS and HR sheep. For physiological metrics (A-C), n = 30 sheep per group; for daily feed intake (D), n = 60 days per group. Data are presented as mean ± SEM. * *P* < 0.05, **** *P* < 0.0001. Statistical significance was determined by a two-tailed Student’s t-test.


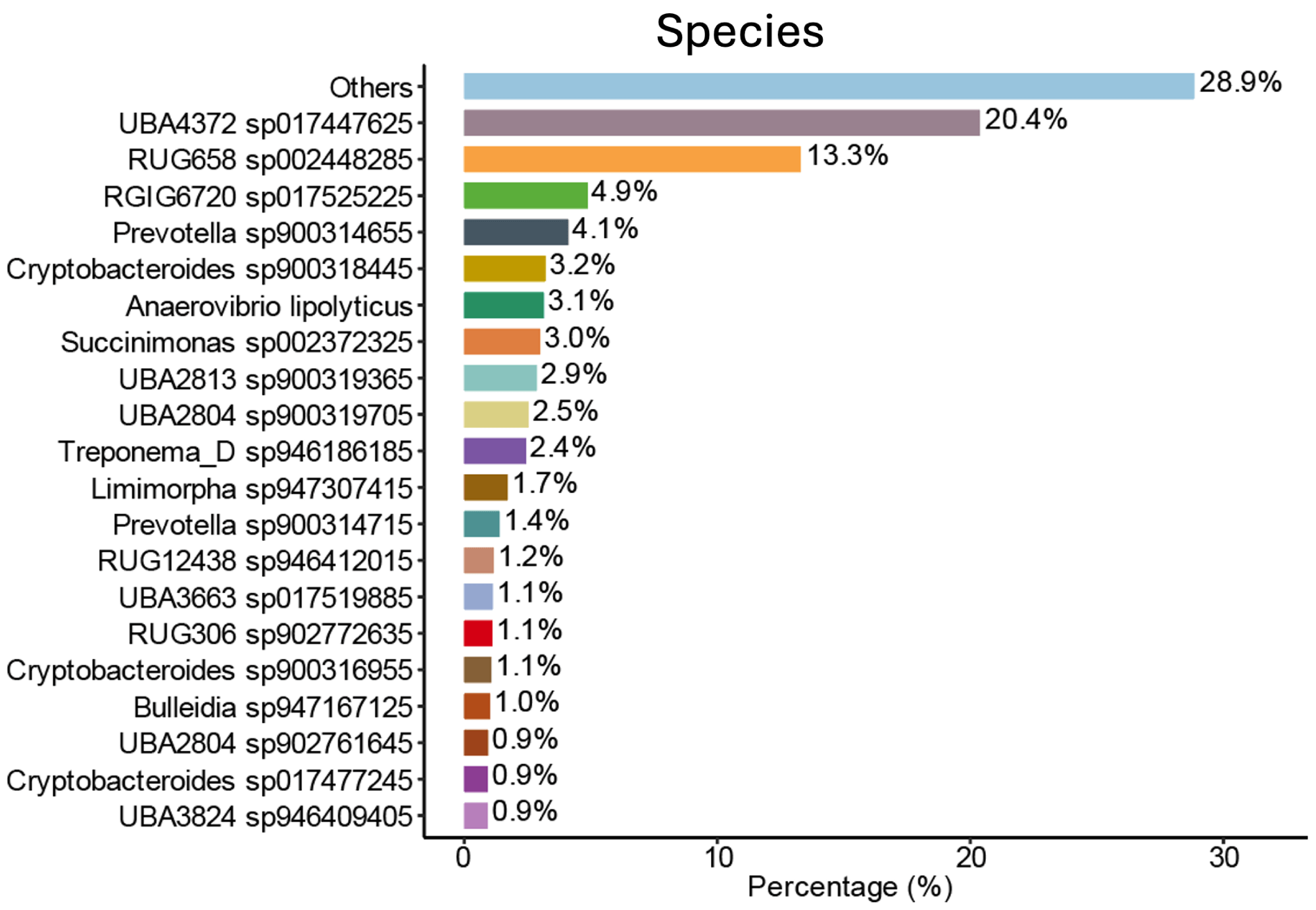


**Fig. S4** Species-level taxonomic distribution of the bacterial community. The bar chart ranks the top bacterial species by relative abundance.


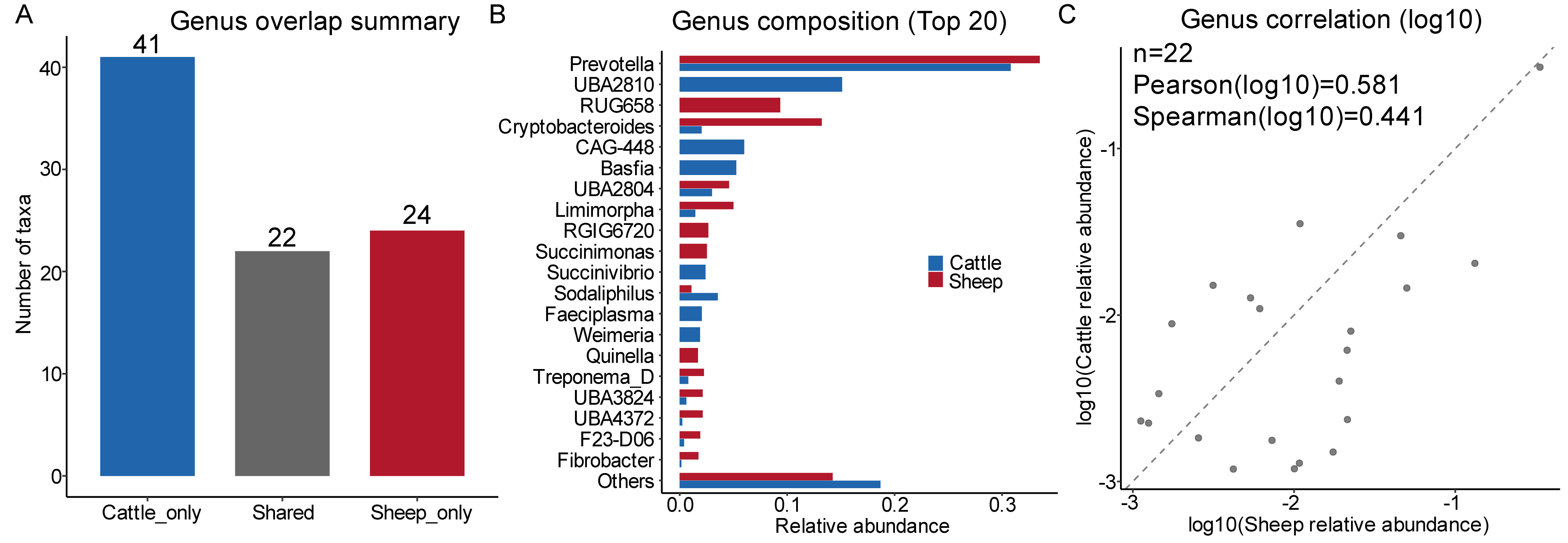


**Fig. S5** Taxonomic overlap, composition, and abundance correlation of the core rumen microbiome in bovine and ovine hosts. (A) Bar plot summarizing the shared (n = 22) and host-unique bacterial genera between cattle and sheep. Core microbiotas were defined as genera with relative abundance > 0.1%. (B)Relative abundance profiles of the top 20 dominant genera across diverse host ecosystems. (C) Pearson correlation analysis of the 22 shared core taxa, based on their log10-transformed relative abundances in bovine and ovine hosts.


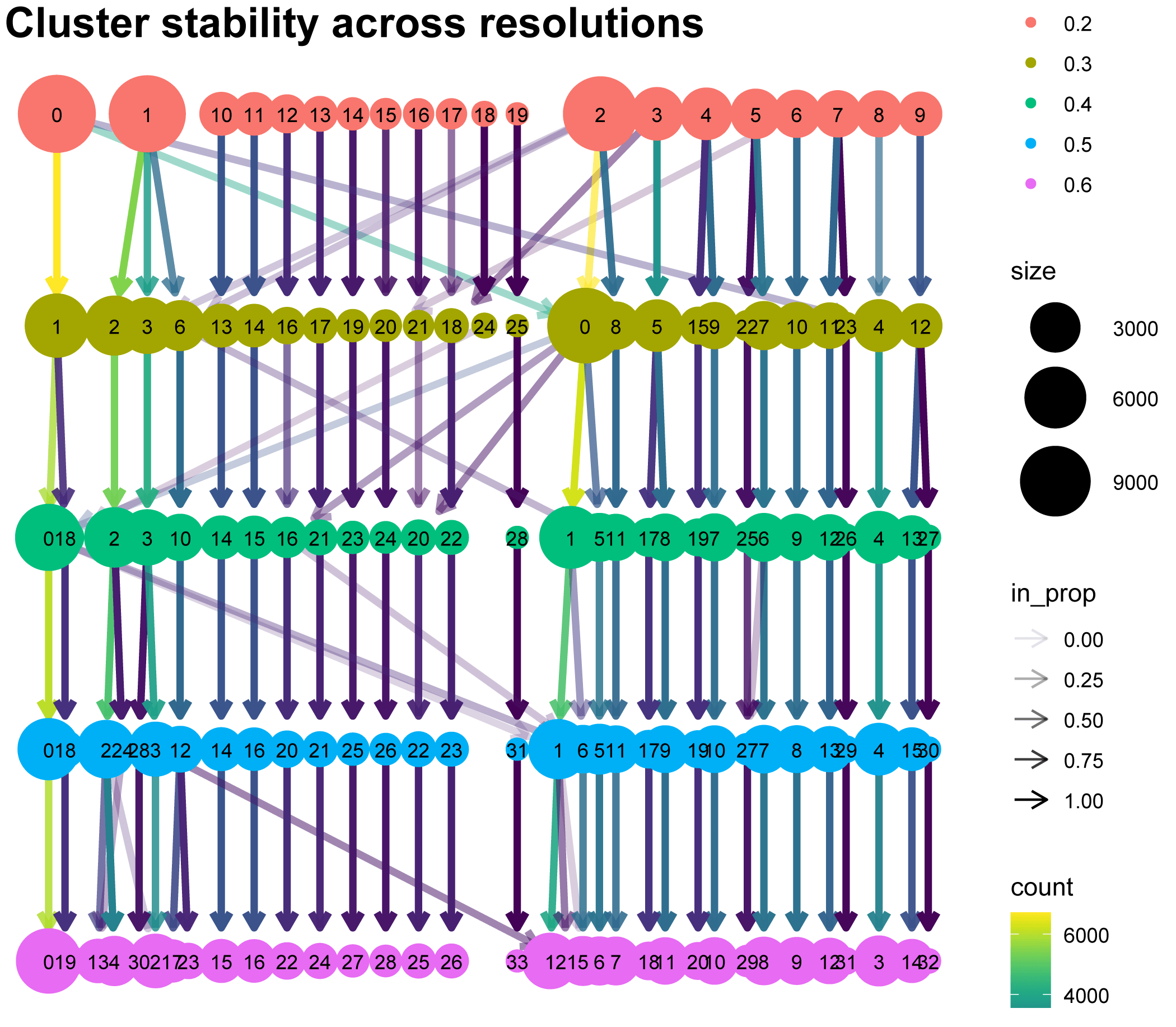


**Fig. S6** Clustering tree analysis evaluating cluster stability across varying resolutions. > The clustree visualization demonstrates the relationship and topological stability of single-cell clusters across a Seurat resolution gradient from 0.2 to 0.6. Nodes represent individual clusters at a specific resolution, with node size proportional to the number of cells within the cluster. Arrows indicate the redistribution of cells between clusters at adjacent resolutions, with color intensity and edge thickness representing the proportion of cells transitioning (in_prop).


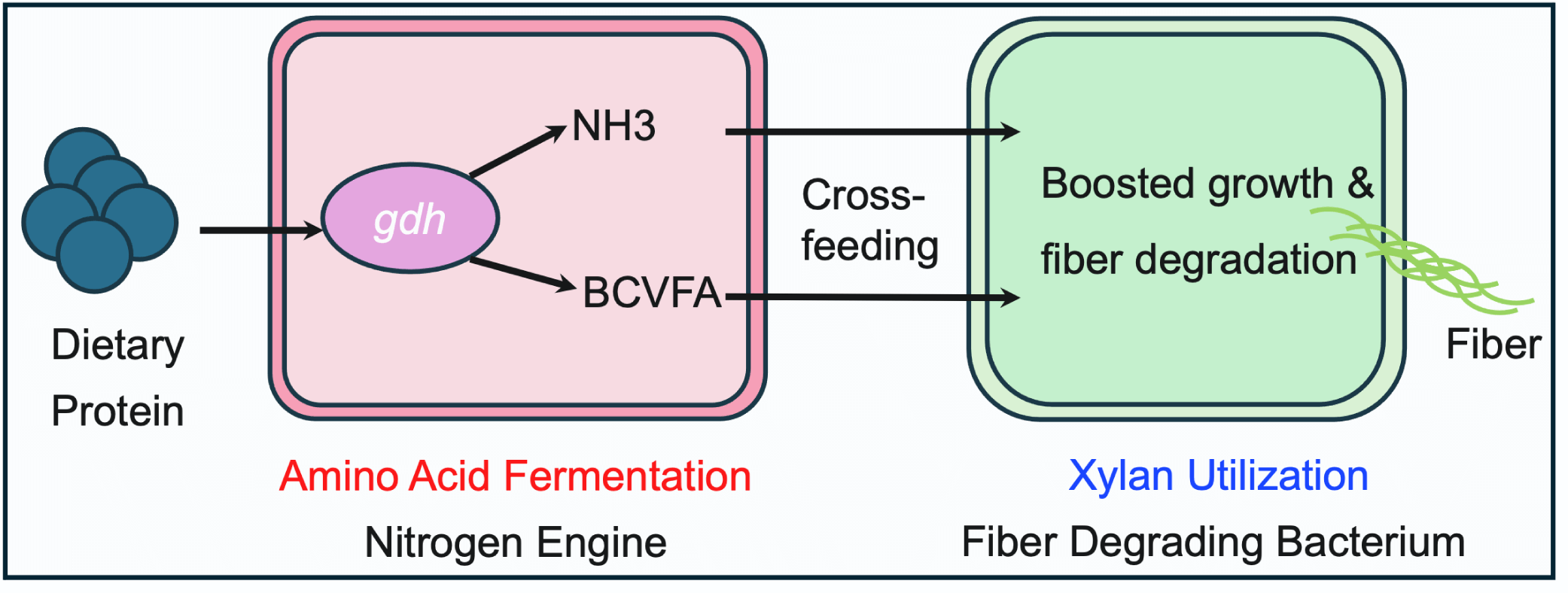


**Fig. S7** Single-cell transcriptomic profiling of rumen bacteria reveals metabolic cross-feeding and division of labor between functional clusters.


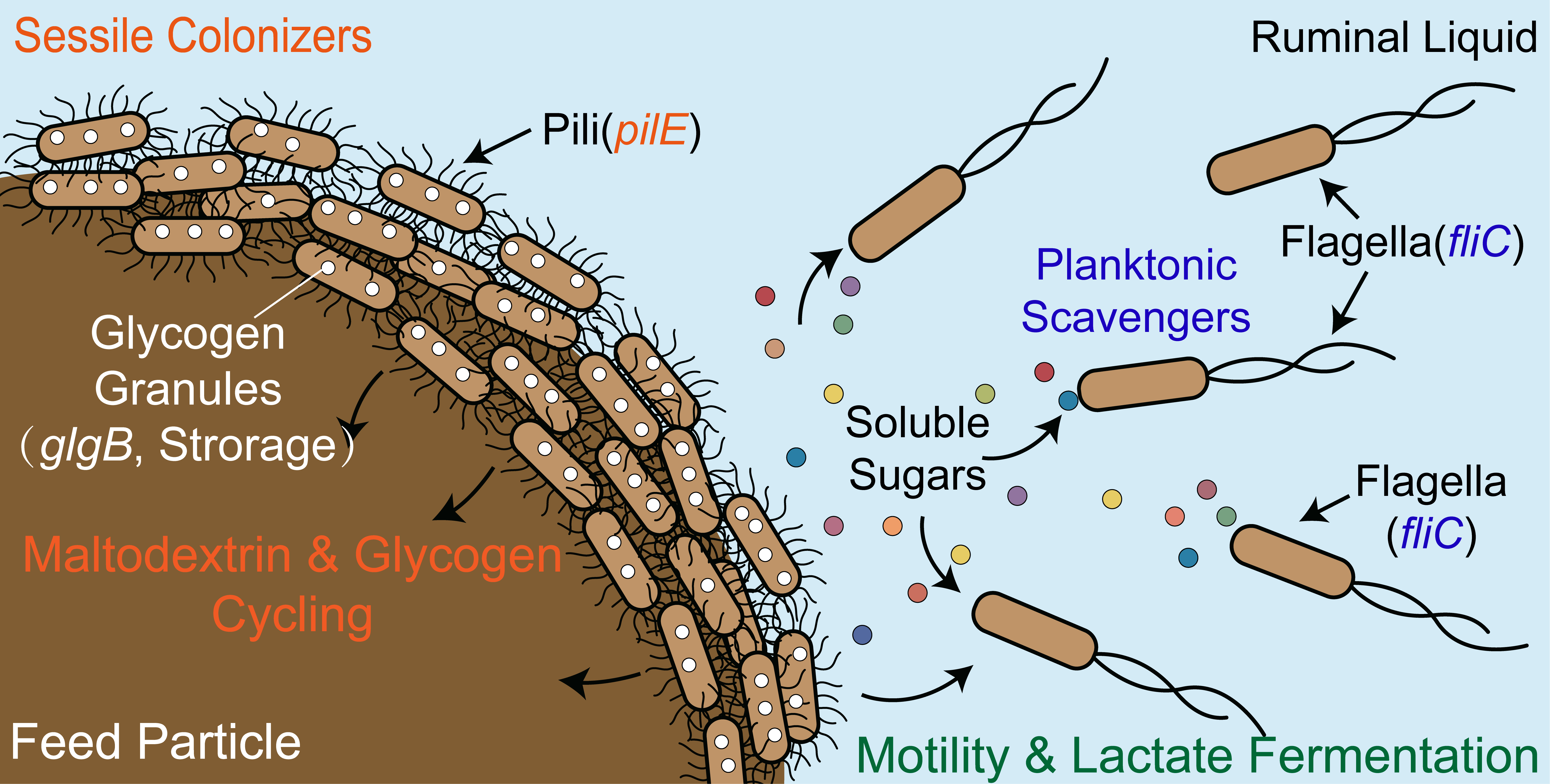


**Fig. S8** Single-cell transcriptomic profiling of rumen bacteria reveals distinct microbial lifestyles (adherent vs. planktonic).


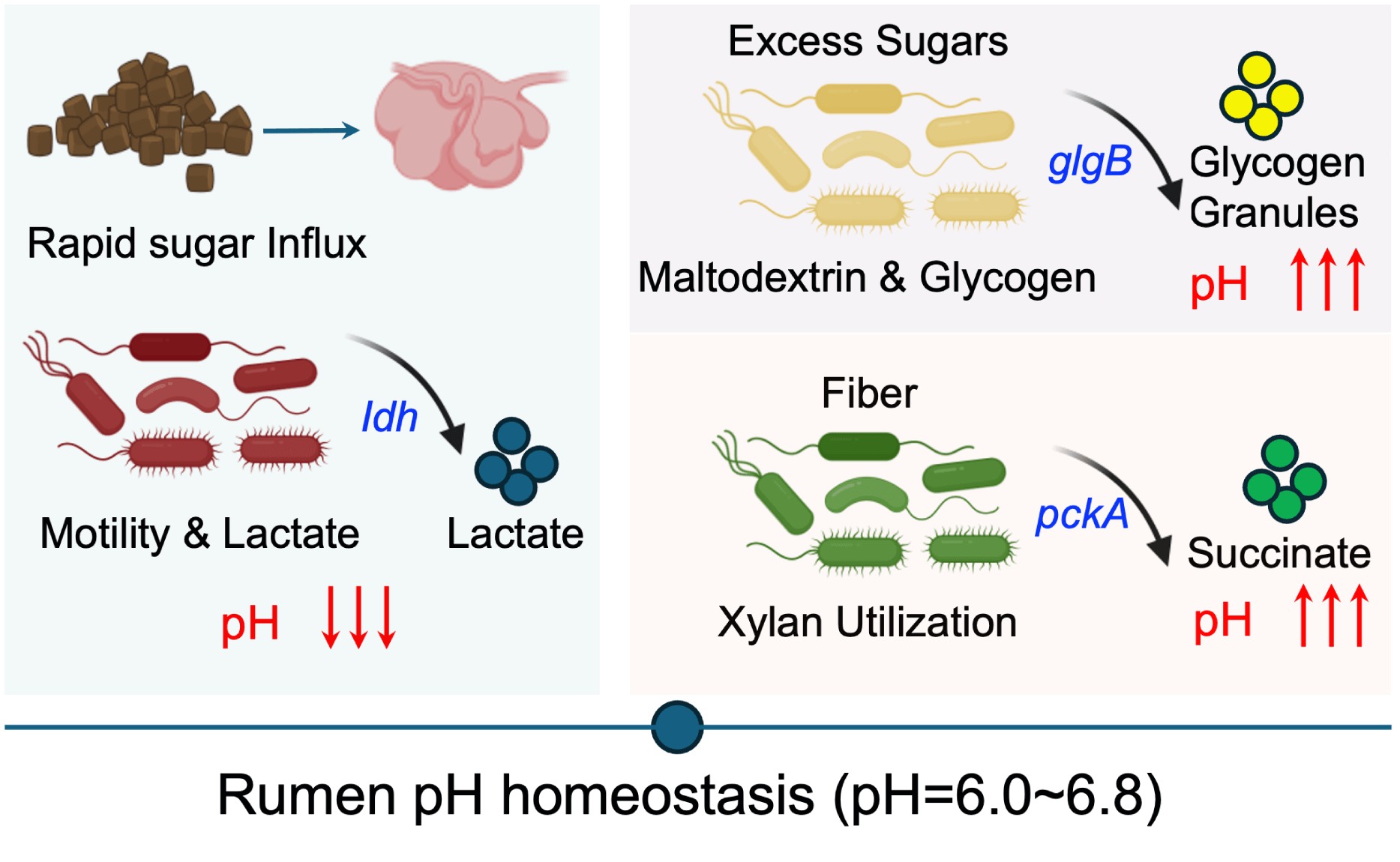


**Fig. S9** Single-cell transcriptomic profiling of rumen bacteria reveals the functional cluster division of labor in the rumen and the maintenance of rumen pH homeostasis.


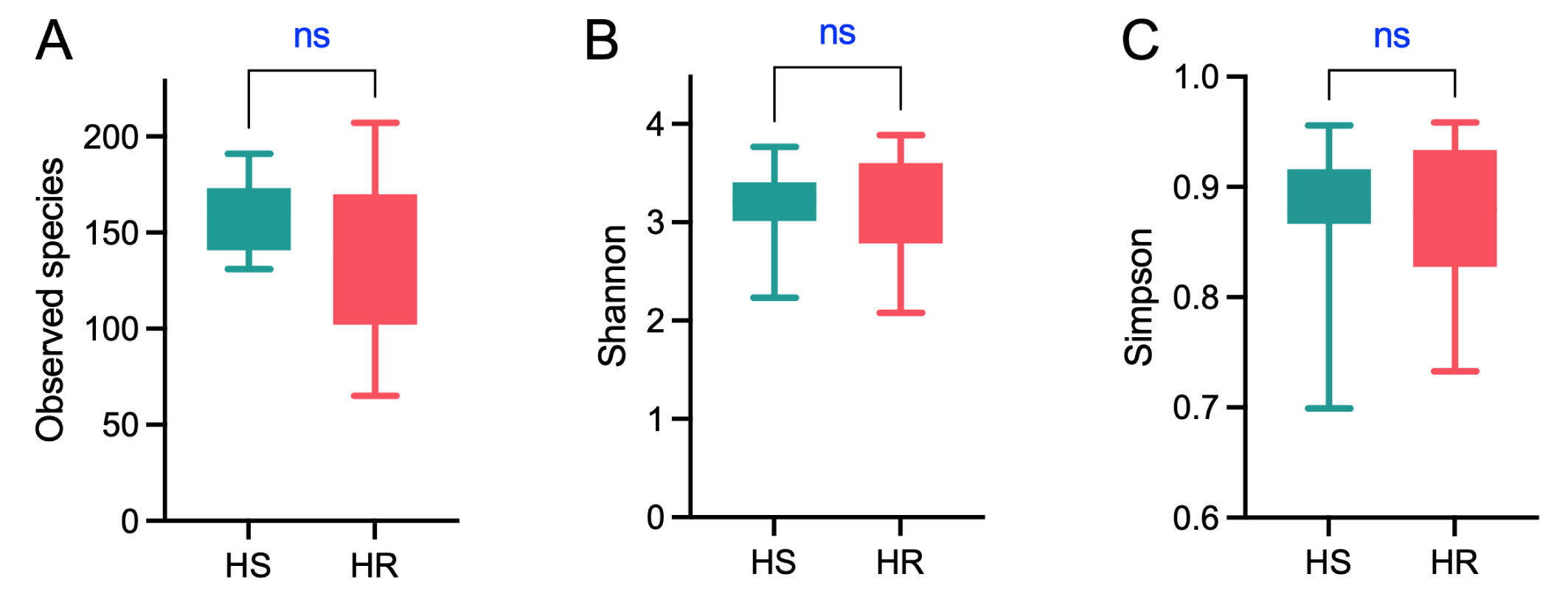


**Fig. S10** Alpha-diversity indices show no significant differences between Heat-Resistant (HR) and Heat-Sensitive (HS) hosts.


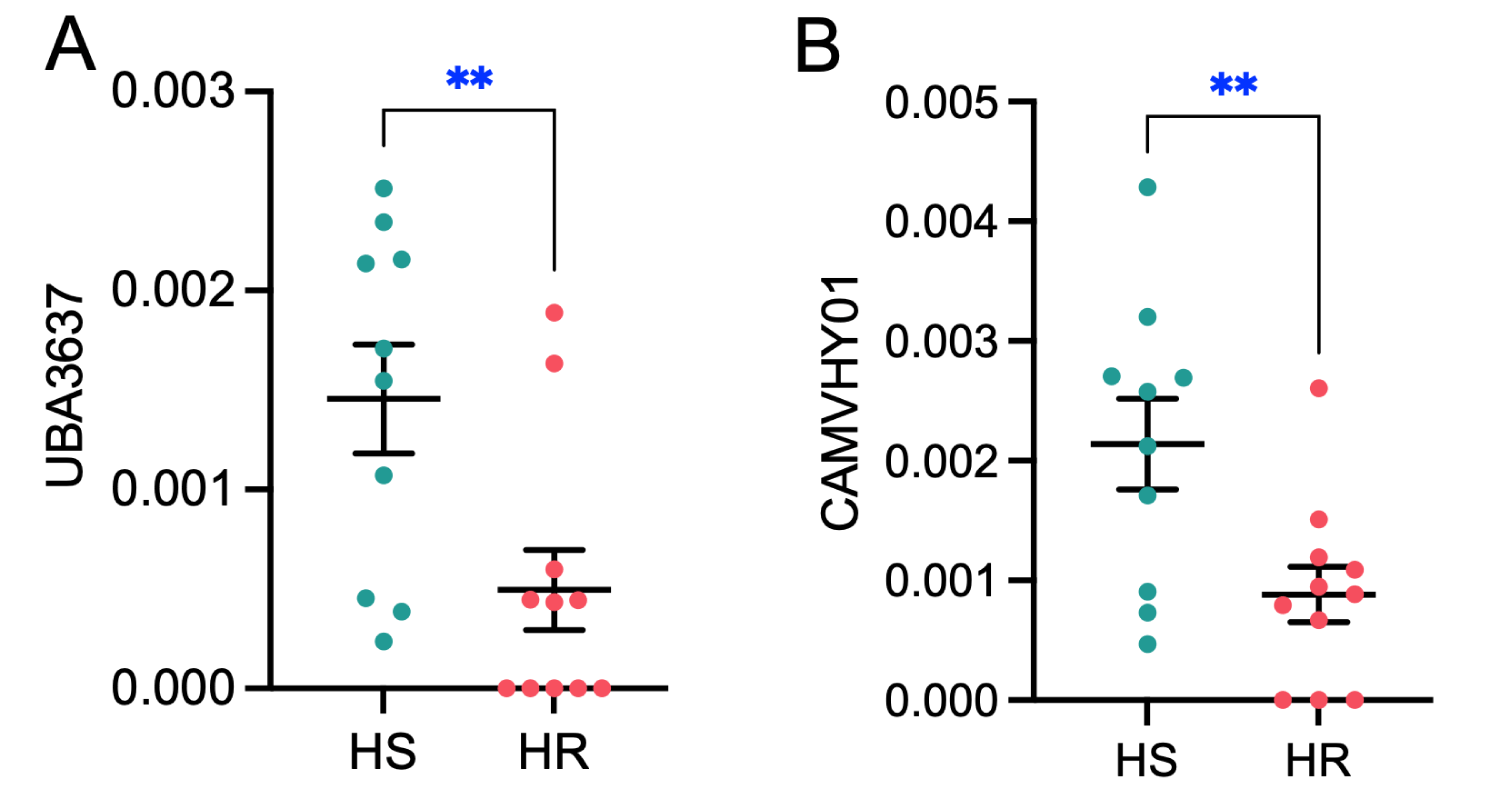


**Fig. S11** Differential abundance of microbial taxa between heat-resistant (HR) and heat-susceptible (HS) hosts at the genus level. Each dot represents one biological sample, ***P* < 0.01, two-tailed t-test.


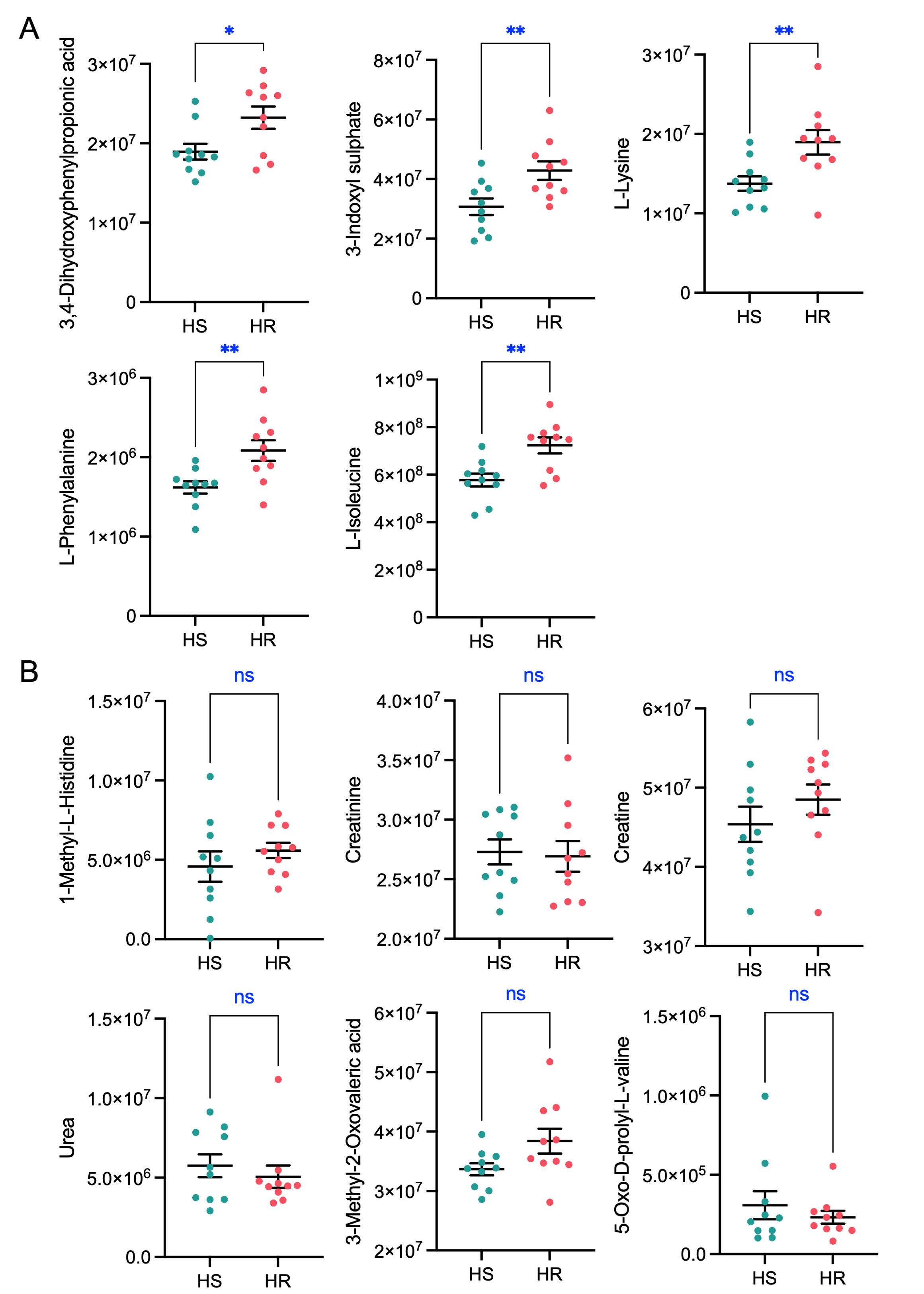


**Fig. S12** Non-targeted serum metabolomic profiling reveals a distinct metabolic state in HR hosts without the activation of hyper-catabolic tissue breakdown. (A) Scatter dot plots demonstrating significantly increased levels of specific serum metabolites in the HR cohort, including 3,4-Dihydroxyphenylpropionic acid, 3-Indoxyl sulphate, essential amino acids (L-Lysine, L-Phenylalanine), and the branched-chain amino acid L-Isoleucine. (B) Quantification of systemic biomarkers associated with skeletal muscle catabolism, nitrogen excretion, and amino acid degradation. The stable levels of myofibrillar degradation proxies (1-Methyl-L-Histidine), along with creatinine, creatine, urea, and downstream branched-chain amino acid degradation intermediates (5-Oxo-D-prolyl-L-valine and 3-Methyl-2-Oxovaleric acid), indicate that the elevated amino acid pool in HR animals is not derived from pathological muscle breakdown. Data are presented as mean ± SEM. * *P* < 0.05, ** *P* < 0.01, ns, not significant (Two-tailed unpaired Student’s t-test).


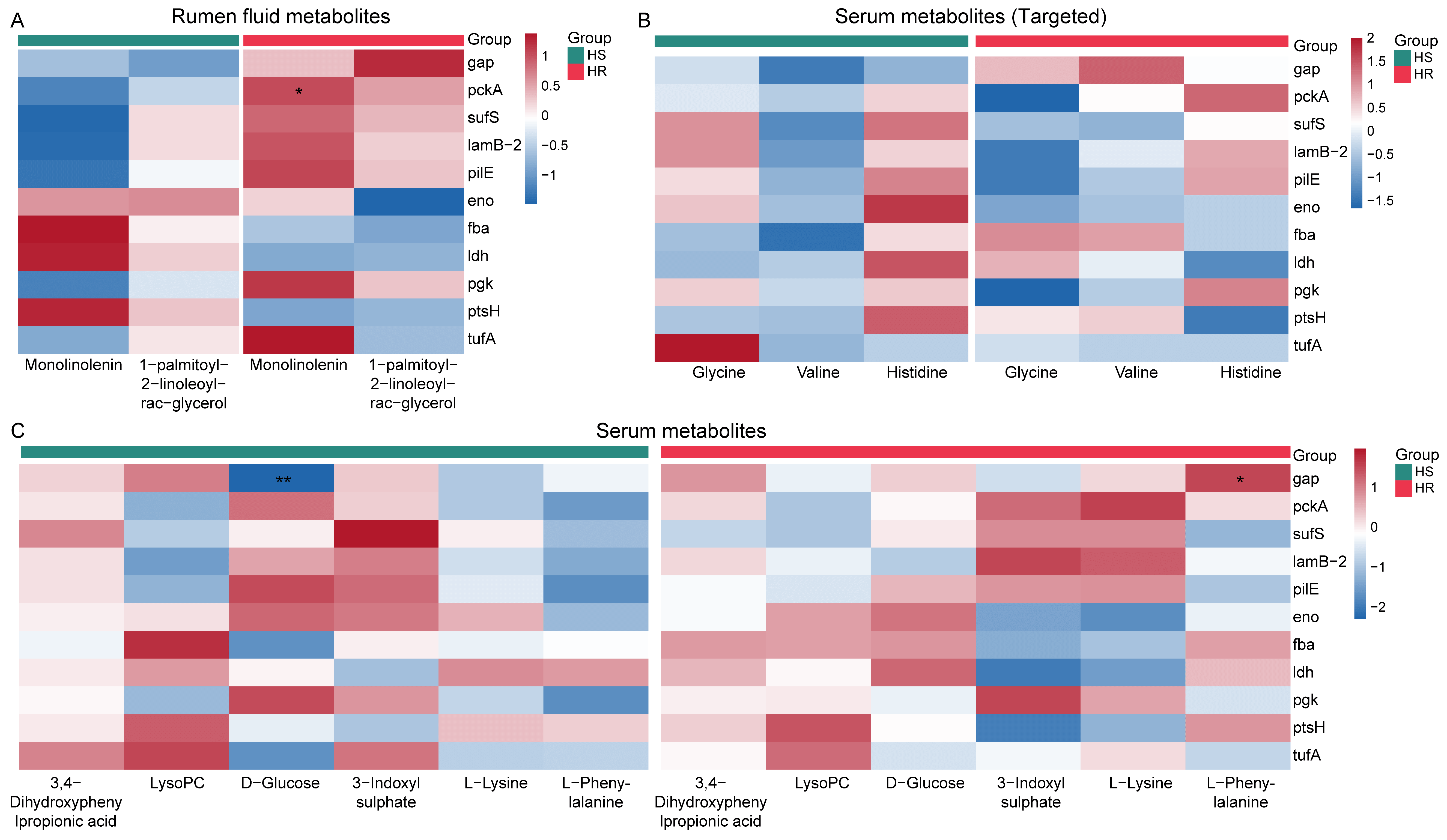


**Fig. S13** Multi-omics correlations between microbial gene expression and host metabolic phenotypes. (A–C) Spearman correlation heatmaps comparing the pseudobulk expression of key microbial glycolytic and gluconeogenic genes per sample with differential metabolites in (A) rumen fluid, (B) targeted serum amino acids, and (C) untargeted serum profiles. Color intensity indicates the Spearman correlation coefficient (rho), with red and blue representing positive and negative correlations, respectively. Asterisks denote statistical significance based on *P*-values (* *P* < 0.05, ** *P* < 0.01).


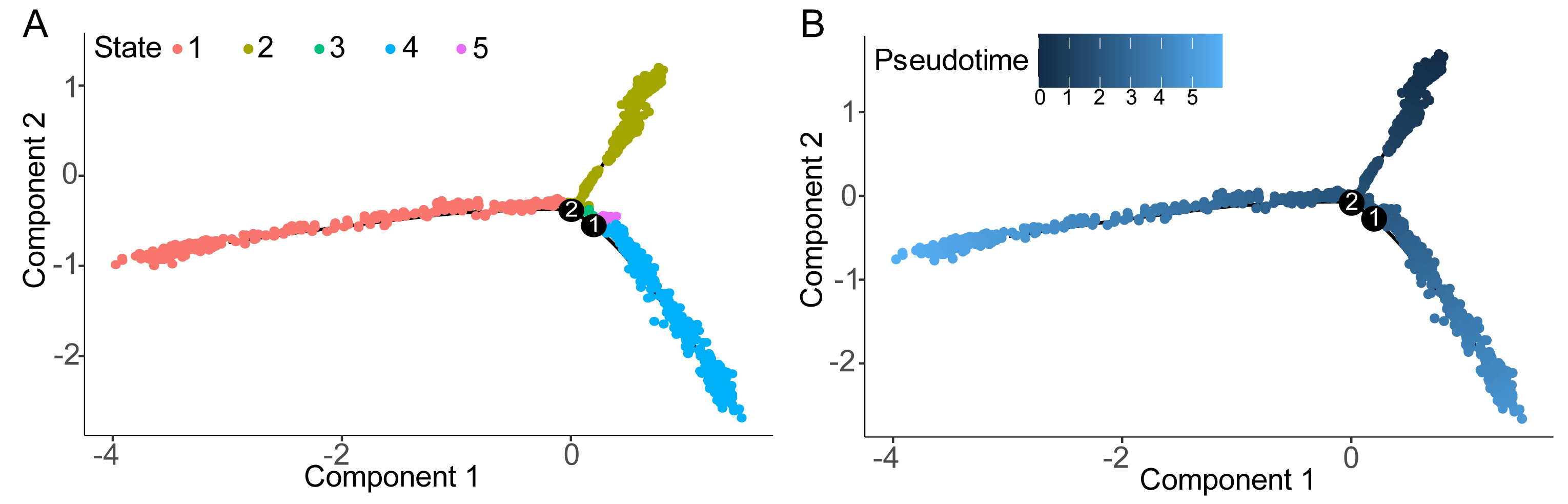


**Fig. S14.** Pseudotime trajectory and state assignment of *Anaerovibrio lipolyticus* subpopulations. (A) The identical trajectory colored by Monocle 2-defined cell states (States 1–5). (B) The single-cell developmental trajectory colored by inferred pseudotime.
